# Genetic and pharmacological activation of Hedgehog signaling inhibits osteoclastogenesis and attenuates titanium particle-induced osteolysis partly through suppressing the JNK/c-Fos-NFATc1 cascade

**DOI:** 10.7150/thno.44793

**Published:** 2020-05-17

**Authors:** Liwei Zhang, Yanjun Yang, Zirui Liao, Qingbai Liu, Xinhuan Lei, Meng Li, Saijilafu, Zunyi Zhang, Dun Hong, Min Zhu, Bin Li, Huilin Yang, Jianquan Chen

**Affiliations:** 1 Department of Orthopaedics, the First Affiliated Hospital of Soochow University, Suzhou, Jiangsu 215006, China; 2 Orthopedic Institute, Medical College, Soochow University, Suzhou, Jiangsu 215007, China; 3 Orthopedic Department, Taizhou Hospital Affiliated to Wenzhou Medical University, Linhai, Zhejiang 317000, China; 4 Institute of Life Sciences, College of Life and Environmental Science, Key Laboratory of Mammalian Organogenesis and Regeneration, Hangzhou Normal University, Hangzhou, Zhejiang 310036, China

**Keywords:** peri-prosthetic osteolysis, titanium particle, Hh signaling, osteoclatogenesis

## Abstract

**Rationale:** Wear particle-induced periprosthetic osteolysis (PPO) is a common long-term complication of total joint arthroplasty, and represents the major cause of aseptic loosening and subsequent implant failure. Previous studies have identified the central role of osteoclast-mediated bone resorption in the pathogenesis of PPO. Thus, therapeutic approaches of inhibiting osteoclast formation and activity are considered to be of great potential to prevent and treat this osteolytic disease. Hedgehog (Hh) signaling has been shown to play an important role in promoting osteoblast differentiation and bone formation. While Hh signaling is also implicated in regulating osteoclastogenesis, whether it can directly inhibit osteoclast differentiation and bone resorption remains controversial. Moreover, its potential therapeutic effects on PPO have never been assessed. In this study, we explored the cell-autonomous role of Hh signaling in regulating osteoclastogenesis and its therapeutic potential in preventing wear particle-induced osteolysis.

**Methods:** Hh signaling was activated in macrophages by genetically ablating *Sufu* in these cells using *LysM-Cre* or by treating them with purmorphamine (PM), a pharmacological activator of Smoothened (Smo).* In vitro* cell-autonomous effects of Hh pathway activation on RANKL-induced osteoclast differentiation and activity were evaluated by TRAP staining, phalloidin staining, qPCR analyses, and bone resorption assays. *In vivo* evaluation of its therapeutic efficacy against PPO was performed in a murine calvarial model of titanium particle-induced osteolysis by μCT and histological analyses. Mechanistic details were explored in RANKL-treated macrophages through Western blot analyses.

**Results:** We found that *Sufu* deletion or PM treatment potently activated Hh signaling in macrophages, and strongly inhibited RANKL-induced TRAP^+^ osteoclast production, F-actin ring formation, osteoclast-specific gene expression, and osteoclast activity *in vitro*. Furthermore, we found that *Sufu* deletion or PM administration significantly attenuated titanium particle-induced osteoclast formation and bone loss *in vivo*. Our mechanistic study revealed that activation of Hh signaling suppressed RANKL-induced activation of JNK pathway and downregulated protein levels of two key osteoclastic transcriptional factors, c-Fos and its downstream target NFATc1.

**Conclusions:** Both genetic and pharmacological activation of Hh signaling can cell-autonomously inhibit RANKL-induced osteoclast differentiation and activity *in vitro* and protect against titanium particle-induced osteolysis *in vivo*. Mechanistically, Hh signaling hinders osteoclastogenesis partly through suppressing the JNK/c-Fos-NFATc1 cascade. Thus, Hh signaling may serve as a promising therapeutic target for the prevention and treatment of PPO and other osteolytic diseases.

## Introduction

Total joint arthroplasty (TJA) has been performed widely to treat arthralgia, joint instability and deformity caused by severe trauma, osteoarthritis, rheumatoid arthritis and osteoporotic fracture. It was estimated that more than one million cases of TJA were performed every year in the U.S.A., and this number was expected to reach two million by the year 2030 1, 2. Although TJA is considered to be one of the most successful operations, aseptic loosening resulting from periprosthetic osteolysis (PPO) is still a common and serious complication, which often leads to implant failure and revision surgery 3-5. While the precise mechanism underlying PPO remains elusive, increasing evidence has pointed to wear particles released from implant surfaces as a major causative factor 6. Wear particles stimulate several different types of cells such as macrophages and T lymphocytes to secrete a variety of proinflammatory chemokines and cytokines 7, 8. These molecules in turn recruit osteoclast precursors, and at the same time act on other target cells, mainly osteoblasts and marrow stromal cells, to promote their expression of receptor activator of nuclear factor-κB ligand (RANKL), a critical regulator of osteoclastogenesis. Upon binding to its receptor RANK on the surface of osteoclasts and their precursors, RANKL triggers activation of multiple intracellular signaling pathways to promote osteoclast differentiation and activity whereas suppressing its apoptosis. As a result, osteoclasts are excessively formed and activated at the peri-implant sites, eventually leading to extensive bone destruction 7, 8. Given the central role of osteoclast-mediated bone resorption in the pathogenesis of PPO, the therapeutic approach inhibiting osteoclast formation and activity is considered to be of great potential to prevent and treat this osteolytic disease. However, clinical studies testing the efficacy of several anti-resorption agents, including bisphosphonate and denosumab, only yielded limited success 9-11. Thus, an alternative agent or approach that can effectively prevent and treat PPO-triggered aseptic loosening is still urgently needed.

In recent years, the therapeutic approaches simultaneously inhibiting bone destruction and promoting bone regeneration have received increasing attention for prevention and treatment of osteolytic diseases 12, 13. Hh pathway, an evolutionarily conserved signaling pathway, plays critical roles in skeletal development and homeostasis, and is regarded as a promising anabolic pathway for treating osteoporosis and promoting bone regeneration 14, 15. This pathway is triggered by binding Hh ligands to their receptor Patched1 (Ptch1), thus relieving its suppression on Smoothened (Smo), a seven-pass transmembrane protein. Active Smo accumulates in the primary cilia, where it reverses the inhibitory effect of the Sufu-containing suppressive complex on formation of the activator form of Gli family transcriptional factors (Gli^A^). Consequently, Gli^A^ is produced and subsequently enters the nucleus to activate transcription of Hh target genes, including *Gli1* and *Ptch1*, two Hh pathway components that are commonly used as readouts of Hh signaling activity 14, 16. Another way to activate Hh signaling is to genetically remove either *Ptch1* or *Sufu* 17, 18. In addition, several pharmacological agents, including purmorphamine, can also directly act on Smo protein to activate signaling independently of Hh molecules 19, 20.

While Hh signaling has been firmly established as an important positive regulator of osteoblast differentiation and bone formation 14, 15, 21, its role in inhibiting osteoclastogenesis and bone resorption still needs to be determined, which has greatly limited its potential application as a therapeutic target with the dual effects of promoting bone formation and suppressing bone resorption in treating osteolytic diseases. In fact, genetic activation of Hh signaling by either *Ptch1* haploinsufficiency or conditional deletion of *Ptch1* in mature osteoblasts was shown to indirectly stimulate osteoclastogenesis through upregulating RANKL expression in osteoblasts both *in vitro* and *in vivo* 22-24. Similarly, some types of tumor cells were reported to secrete Hh ligands to activate Hh signaling in osteoblasts to stimulate their production of RANKL, which aberrantly increased the number of osteoclasts to cause metastatic osteolysis 25, 26. The above *in vitro* and *in viv*o studies have clearly demonstrated that Hh signaling can promote osteoclastogenesis via a non-cell-autonomous mechanism under physiological and certain pathological conditions. In contrast, the cell-autonomous role of Hh signaling in osteoclastogenesis remains unclear. While a few *in vitro* studies did explore the direct effect of Hh pathway activation on osteoclast differentiation, these studies reported inconsistent results 27-32, none of which was validated *in vivo*. Interestingly, a recent genetic study revealed that conditional ablation of *Ihh* in limb mesenchymal cells, which potentially disrupted Hh signaling in both osteoblastic and osteoclastic lineage cells, led to increased osteoclast formation 17. Although the exact mechanism underlying this inhibitory effect of Ihh signaling on osteoclastogenesis remains to be elucidated, this study raised the possibility that Hh signaling can cell-autonomously inhibit RANKL-induced osteoclast differentiation despite its stimulatory effect on RANKL expression in osteoblasts. However, direct evidence to support such a suppressive role of Hh activation in osteoclastogenesis is still lacking. Given this uncertainty, it remains to be determined whether activation of Hh signaling can be utilized to prevent osteolytic diseases, such as wear particle-induced osteolysis.

In this study, we explored the cell-autonomous role of Hh signaling in regulating osteoclastogenesis and its therapeutic potential in preventing wear particle-induced osteolysis using both genetic and pharmacological approaches. Our results demonstrated that activation of Hh signaling either by conditionally deleting *Sufu* (a key negative regulator of Hh signaling) in osteoclast precursors or by treatment with purmorphamine (a pharmacological activator of Smo protein) inhibited RANKL-stimulated osteoclast differentiation *in vitro* and attenuated Ti particle-induced osteoclastogenesis and bone loss *in vivo*. Mechanistically, we found that Hh signaling directly inhibited osteoclast differentiation through suppressing the JNK/c-Fos-NFATc1 cascade. Thus, our study revealed a previously unrecognized role of Hh signaling in inhibiting osteoclastogenesis and provided the first preclinical evidence to support activating Hh pathway as a potential therapeutic approach to preventing and treating PPO and aseptic loosening.

## Materials and Methods

### Antibodies and other reagents

Rabbit primary antibodies against p-p65 (#3033, 1:1000), AKT (#9272, 1:1000), p-AKT (#9271, 1:1000), JNK (#9252, 1:1000), p-JNK (#4668, 1:1000), p38 (#8690, 1:1000), ERK (#4695, 1:1000), p-ERK (#4370, 1:1000), and GAPDH (#5174, 1:2000) were obtained from Cell Signaling Technology (Cambridge, MA, USA). Rabbit primary antibodies for p-p38 (#ab4822, 1:1000), NFATc1 (#ab25916, 1:1000) and c-fos (#ab190289, 1:1000) were purchased from Abcam (San Francisco, CA, USA). Rabbit primary antibodies against total p65 (#10745-1-AP,1:1000), Sufu (#26759-1-AP,1:1000), and PCNA (#10205-2-AP,1:1000) were purchased from proteintech (Wuhan, Hubei, China). HRP-conjugated anti-rabbit secondary antibody (#7074, 1:2000) was provided by Cell Signaling Technology (Cambridge, MA, USA). Recombinant mouse macrophage-colony stimulating factor (M-CSF) and receptor activator for nuclear factor-κB ligand (RANKL) were purchased from R&D Systems (Minneapolis, MN, USA), and used at 30 ng/ml and 50 ng/ml, respectively. Purmorphamine (PM) was purchased from Selleck Chemicals (Houston, TX, USA) and dissolved in DMSO with a concentration of 40 mM. FITC-phalloidin and DAPI were purchased from MesGen Biotech (Shanghai, China), and used at 1:100 and 1:2000 dilutions, respectively. The α-minimum essential medium (α-MEM), fetal bovine serum (FBS), penicillin/streptomycin (P/S), and 2.5% trypsin were obtained from Gibco-BRL (Sydney, Australia). RIPA buffer was provided by Beyotime Biotechnology (Shanghai, China). cOmplete™ protease and PhosSTOP™ phosphatase inhibitors were purchased from Roche (Indianapolis, IN, USA). Cell Counting Kit-8 (CCK-8) was supplied by Dojindo laboratories (Mashiki-machi, Kumamoto, Japan).

### Mouse strains

Mice carrying *Sufu* conditional allele (*Sufu^flox^*) were generated by flanking Exon 7 with two repeated *LoxP* sites as described previously 18. *LysM*-Cre, a knockin mouse line expressing Cre recombinase under the control of the endogenous M lysozyme locus, was previously reported 33 and obtained from the Jackson Laboratories (Bar Harbor, ME, USA). To conditionally delete *Sufu* in monocyte/macrophage lineage cells,* LysM-Cre^+/+^; Sufu^flox/+^*mice were intercrossed to obtain progenies carrying two copies of both* LysM-Cre* and *Sufu^flox^* alleles (*LysM-Cre^+/+^; Sufu^flox/flox^,* hereafter referred to as *Sufu^CKO^).* Their gender-matched littermates with *LysM-Cre^+/+^* genotype were used as controls. For experiments involving only wildtype mice, 8 to 10-week-old male C57BL/6 mice were used. All mice were raised in the specific pathogen-free (SPF) animal facility at Laboratory Animal Center of Soochow University. All experimental protocols involving the use of animals were approved by the Ethics Committee of the First Affiliated Hospital of Soochow University (#201810A044).

### Preparation and culture of mouse bone marrow-derived macrophages (BMMs)

To prepare mouse primary BMMs, total bone marrow cells were isolated from the femur and tibia of 8 to 10-week-old mice as previously described 34, 35. Isolated bone marrow cells were then plated in 10 cm cell culture dishes, and cultured in BMM maintenance medium (α-MEM containing 10% FBS, 1% P/S, and 30 ng/ml recombinant mouse M-CSF) for 24 h. Following complete aspiration of old media, cultures were briefly rinsed with DPBS to remove non-adherent cells. The remaining attached BMMs were further expanded in BMM maintenance medium for additional 2-3 days until they reached nearly 100% confluence. Confluent BMMs were subsequently trypsinized and re-seeded in cell culture plates at a density of 2×10^4^ cells/cm^2^ unless otherwise indicated. All cell cultures were maintained in a 37 °C humidified incubator with 5% CO_2_ (v/v), and medium was changed every other day.

### *In vitro* osteoclast differentiation assays

For *in vitro* osteoclast differentiation, BMMs were seeded in a 24-well plate (for TRAP or F-actin ring staining) or 6-well plate (for protein or RNA analysis) at the density of 2×10^4^ cells/cm^2^, and incubated in BMM maintenance medium for 18 h. Osteoclastic differentiation of BMMs was then induced with differentiation medium (BMM maintenance medium supplemented with 50 ng/ml recombinant mouse RANKL) for 5-6 days with the medium changed every 2 days. In experiments involving PM treatment, vehicle or different concentrations of PM (0.5, 1, or 2 μM) was included in the differentiation medium. At the end of osteoclastic induction, status of osteoclast differentiation was evaluated by TRAP staining, F-actin ring immunofluorescence, qPCR, or Western blot analyses as indicated.

To assess the effect of PM on protein expression of key osteoclastic transcriptional factors during the course of osteoclastogenesis, BMMs were seeded and cultured as described above. Afterwards, BMMs were stimulated with 50 ng/ml RANKL in the presence of vehicle or 2 μM PM for 0,1 or 3 days. At each time point, cells were harvested for protein extraction.

To determine the effect of PM on RANKL-mediated activation of intracellular signaling pathways, a higher number (4×10^5^ cells/well) of BMMs were planted in 6-well plates, and cultured in BMM maintenance medium for 18 h. BMMs were then pretreated with vehicle (DMSO) or 2 μM PM for 4 h, followed by stimulation with 50 ng/ml RANKL for 0, 5, 15, 30 or 60 min. At each time point, cells were harvested for isolation of total RNA, total protein or cytoplasmic/nuclear protein.

### Cell viability assay

The effect of PM on viability of BMMs was determined using Cell Counting Kit-8 (CCK-8) (Dojindo Laboratories, Japan) according to the manufacturer's instruction. Briefly, BMMs were plated into 96-well plates at a density of 8×10^3^ cells/well in triplicate and cultured in BMM maintenance medium for 18 h. Afterwards, the cells were treated with vehicle (DMSO) or different concentrations of PM (0.0625, 0.125, 0.25, 0.5, 1, 2, 4, 8, 16 and 32 μM) for 48, 72 and 96 h. At each of these time points, 10 μl CCK-8 solution was added to each well, and the plates were then incubated for 1 h at 37 °C. The optical density at 450 nm (OD_450_) was measured by an ELX800 absorbance microplate reader (Bio-Tek, Winooski, USA). The OD_450_ values were plotted against the corresponding PM concentrations using GraphPad Prism.

### TRAP staining

TRAP staining of cultured cells was performed as we previously described 36. Briefly, cells were washed once with phosphate-buffered saline (PBS), and then fixed with 4% paraformaldehyde (PFA) for 10 min. After being rinsed twice with PBS, cells were incubated with tartrate buffer at 37 °C for 30 min. Subsequently, cells were stained with TRAP staining solution (tartrate buffer containing naphthol AS-BI phosphate and pararosaniline chloride) at 37 °C for 1 h in darkness. Images of stained cells were then acquired using Axiovert 40 CFL microscope (Carl Zeiss Microscopy, Thornwood, NY). TRAP-positive cells with more than three nuclei were considered as mature osteoclasts. The number and relative size of mature osteoclasts in each well were quantified by Image J software (v.1.51, National Institutes of Health, USA). A minimum of 3 wells were analyzed for each group.

### Quantitative PCR (qPCR)

Total RNA was isolated from whole cells with Total RNA Extractor (Sangon Biotech, Shanghai, China) in accordance with the instructions provided in the manual. 500 ng of total RNA was reverse-transcribed into cDNA using HiScript II Q RT SuperMix (Vazyme, Nanjing, China). PCR reaction was set up in triplicates in a 20 µl volume comprised of 10 µl SYBR Green qPCR Mix (Novoprotein, Shanghai, China), 7.5 µl ddH_2_O, 2 µl cDNA, and 0.5 µl of primer pairs. Real time PCR was performed on the CFX Connect™ Real-Time PCR Detection System (Bio-Rad Laboratories, CA, USA), with the following cycling conditions: 40 cycles of denaturation at 95 °C for 10 s and amplification at 60 °C for 30 s, followed by melting curve analysis from 65 °C to 95 °C at 0.5 °C/step. Expression of each target gene was normalized by 18S ribosomal RNA. The relative changes in mRNA level were analyzed by 2^-ΔΔCT^ method as previously reported 37. The nucleotide sequences for the primers used in this study are provided in Table S1.

### Western Blot analyses

Total protein was extracted from cells using 1×RIPA buffer supplemented with protease and phosphatase inhibitors as we previously described 35. Cytoplasmic and nuclear protein extracts were isolated using a Nuclear and Cytoplasmic Protein Extraction Kit (Beyotime Biotechnology, Shanghai, China) according to the manufacturer's instructions. Concentrations of protein samples were measured using a BCA Protein Assay Kit (Beyotime Biotechnology, Shanghai, China) as per instructions. Thirty-microgram (30 μg) protein lysates were separated on 10% SDS-PAGE gels by electrophoresis, and subsequently transferred onto 0.2 μm NC membranes (GE Healthcare, Freiburg, Germany) by electroblotting. After being blocked with 5% nonfat milk in 1×TBST for 1 h, the blots were incubated with primary antibodies at 4 °C overnight. The next day, the membranes were incubated with HRP-conjugated secondary antibodies at RT for 1 h. Afterwards, the immunoreactivity was revealed using the chemiluminescence reagent (Millipore, Billerica, MA) and then imaged with ChemiDoc™ Touch Imaging System (Bio-Rad Laboratories, CA, USA). Quantification of the band intensity was performed using Image J software.

### Staining of F-actin rings

F-actin ring formation was visualized by phalloidin staining as previously described with minor modifications 38. In brief, BMMs were washed with PBS prior to fixation with 4% paraformaldehyde for 10 min at RT. The fixed cells were then permeabilized with 0.1% Triton X-100 for 5 min, followed by incubation with 3% BSA in PBS for 20 min. Subsequently, cells were stained with FITC-conjugated phalloidin (1:100) at RT in darkness for 20 min. After washing, cells were stained with DAPI (1:2000) for 5 min, rinsed with PBS, and then mounted with anti-fade mounting medium (Beyotime Biotechnology, Shanghai, China). Fluorescent staining was observed and photographed using the Axiovert 40 CFL fluorescent microscope installed with Zeiss ZEN software (Carl Zeiss Microscopy, Thornwood, NY). The number and relative size of F-actin rings in each group were measured using Image J software (National Institutes of Health, MD, USA). A minimum of 3 wells per group were analyzed.

### Bone resorption assay

The effect of PM on bone-resorbing activity of osteoclasts was evaluated using bovine bone slices as previously reported 39. In brief, bone marrow cells were seeded in 6-well plates at a density of 2×10^4^ cells/cm^2^ and stimulated with 30 ng/ml M-CSF and 50 ng/ml RANKL for 3 days. After trypsinization, differentiating osteoclasts were re-seeded onto bovine bone slices placed in individual wells of a 96-well plate at a density of 8×10^3^ cells/well, and further cultured in osteoclast differentiation medium containing 0, 0.5, 1 or 2 μM PM until they became fully differentiated. Subsequently, bone slices were treated with 5% sodium hypochlorite for 5 min to eliminate any attached cell, and then washed twice with ddH_2_O. After that, resorption pits on bone slices were visualized and imaged by a FEI Quanta 250 scanning electron microscope (Hillsboro, OR, USA). The number and relative resorption area were analyzed using the Image J software (National Institutes of Health, MD, USA).

### Characterizations of Ti particles

Pure titanium (Ti) particles (>93% in purity and < 20 μm in diameter) were supplied by Alfa Aesar (#000681, Heysham, UK). Characterization of their morphology and size has been previously reported 40, 41. The sizes of these particles were in the range of 0.9-5.7 μm and their average size was 3.3 μm 40, 41. To remove endotoxins, the particles were baked at 180 °C for 6 h, followed by immersing in 75% ethanol for 48 h as previously described 42. After being washed four times with sterile ultra-pure water, Ti particles were re-suspended in sterile PBS at a concentration of 1 g/ml for further use. Morphology of Ti particles before and after being baked for 6 h at 180 °C was observed and imaged by a scanning electron microscope (FEI Quanta 250, Hillsboro, OR, USA). The effect of the baking treatment on the phase composition of Ti particles was analyzed by X-ray diffraction (XRD) using a SmartLab 9kW X-ray diffractometer (Rigaku, Tokyo, Japan) with CuKa radiation (40Kv, 150mA) in a 2θ range of 5-90° at a step size of 0.02° and a scanning speed of 6°/min.

### Ti particle-induced murine calvarial osteolysis model

Two independent sets of experiments were performed to determine the potential effect of activation of Hh signaling on Ti particle-induced osteolysis. In the first experiment, 10-week-old male *LysM-Cre^+/+^*(Control) mice or *LysM-Cre^+/+^; Sufu^flox/flox^* (Sufu^CKO^) mice were assigned to the following groups (n=5 mice per group): control mice with sham operation (Sham-Ctrl group), *Sufu^CKO^* mice with sham operation (Sham-*Sufu^CKO^* group), control mice with Ti implantation (Ti-Ctrl group), and *Sufu^CKO^* mice with Ti implantation (Ti-*Sufu^CKO^* group). In the second experiment, twenty-eight C57BL/6 mice (male, 10-week-old) were randomly separated into 4 groups (n= 7 mice per group): sham operation+vehicle treatment group (Sham group), Ti implantation+vehicle treatment group (vehicle group), Ti implantation+ 2.5 mg/kg PM treatment group (low-PM group), and Ti implantation+ 10 mg/kg PM treatment group (high-PM group). In both sets of experiments, Ti particle-induced calvarial osteolysis model was established in all Ti-implantation groups. Briefly, mice were anesthetized by intraperitoneal injection of ketamine and xylazine following the standard protocol. Following anesthesia, skin on the calvariae was shaved, disinfected and then incised with a sharp scalpel along the middle line. Subsequently, 30 mg of Ti particles (30 μl) were evenly spread on the surfaces of the bilateral parietal bones, followed by closure of the surgical skin incision. In all sham-operated groups, mice were subjected to the same above surgical procedures except that equal volume of pure PBS (30 μl) was used to replace Ti particle suspension. For pharmacological activation of Hh signaling in mice, PM was diluted in a vehicle solution consisting of 2% DMSO, 30% PEG 300 and 5% Tween 80, and subcutaneously injected to mice in low- and high-PM groups in the center of the calvariae at a dosage of 2.5 mg/kg and 10 mg/kg, respectively. The PM dosage of 10 mg/kg used in this study was converted from a dose of 5 mg/kg previously used in rats based on relative body surface area of rats and mice 43, 44. As control, the same amount of vehicle solution was given to mice in sham and vehicle groups. Both vehicle and PM were administered once daily for consecutive 14 days. Two weeks after the surgery, all mice were sacrificed, and their calvariae were isolated and fixed with 4% PFA for 2 days. After fixation, calvarial samples were washed three times with PBS and then stored in 75% ethanol at 4 °C until further analysis.

### Micro-computed Tomography (μCT) analyses

For μCT analysis, implanted-Ti particles were carefully removed from each calvaria to avoid metal artifacts. After that, the specimens were scanned with a SkyScan 1176 scanner (Bruker, Aartselaar, Belgium) at an isometric resolution of 9 μm with X-ray energy source set at 50 kV and 500 μA. Raw data obtained from μCT scanning were reconstructed with NRecon software and then reoriented with Dataviewer software (Bruker, Aartselaar, Belgium) to generate two-dimensional coronal images of μCT slices. For quantitative analysis, 100 μCT slices in the center of each calvaria were used, and region of interest (ROI) was selected as previously described 13. CTAn software (SkyScan, Aartselaar, Belgium) was utilized to generate three-dimensional (3D) images and calculate the percentage of bone volume out of total tissue volume (BV/TV). 3D μCT images were visualized using Mimics v10.01 software (Materialise, Leuven, Belgium). The number of pores and percentage of porosity were counted by Image J software.

### Histological and histomorphometric analyses

Following μCT scanning, calvariae were decalcified in 14% EDTA for 14 days. The EDTA solution was changed daily. After decalcification, calvarial samples were dehydrated in increasing concentrations of ethanol, cleared in xylene, and embedded in paraffin prior to being sectioned coronally at 6 μm with a Leica microtome (Leica Biosystems, Buffalo Grove, IL, USA). For assessing the degree of bone erosion and the number of osteoclasts, coronal sections at the middle level were subjected to standard H&E and TRAP staining, respectively. Images of stained sections were captured using the Axiovert 40 CFL microscope with Zeiss ZEN software (Carl Zeiss Microscopy, Thornwood, NY). The regions containing the discontinuous and non-osseous tissues were considered as the osteolytic areas, and selected for histomorphometric analyses. The proportion of eroded surface as well as the number of TRAP-positive multinucleated osteoclasts in the selected regions of each group were quantified by Image J software.

### Statistical analyses

Data were presented as average ± standard deviation (S.D.) from at least three independent samples. SPSS 19.0 software (SPSS Inc, Chicago, IL, USA) was used for statistical analyses. Statistical differences between two groups were determined by two-tailed Student's t-test. A *p* value less than 0.05 was considered to be statistically significant.

## Results

### *LysM-Cre*-mediated ablation of *Sufu* activated Hh signaling in macrophages

**Figure 1:**
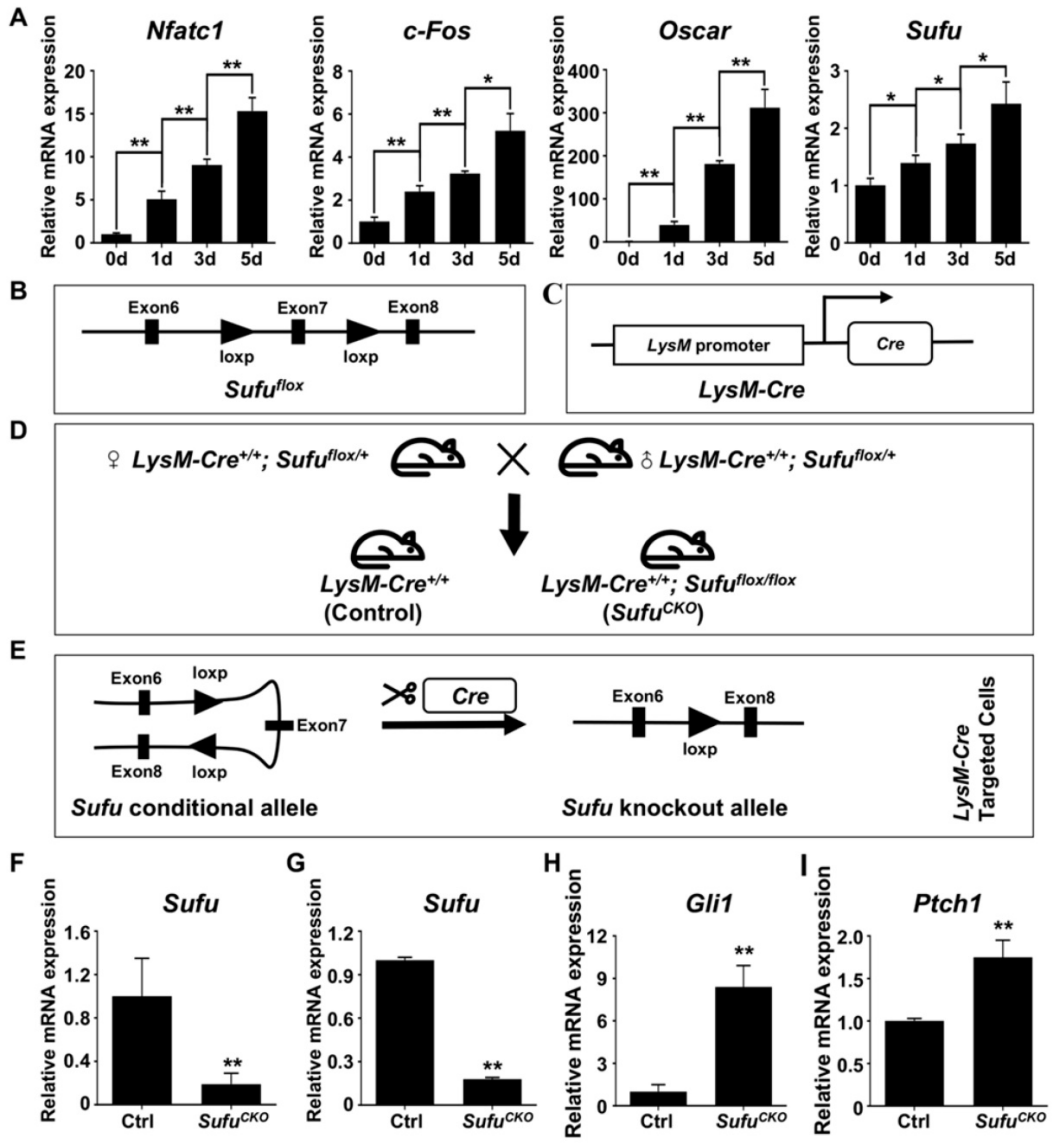
***LysM-Cre*-mediated ablation of *Sufu* activated Hh signaling in macrophages.** (**A**) qPCR analyses of relative mRNA levels of *Nfatc1, c-Fos, Oscar,* and* Sufu* in BMMs at 0, 1, 3, and 5 days after RANKL stimulation. Expression of each target gene was normalized by 18S ribosomal RNA. The relative changes in mRNA level were analyzed by 2^-ΔΔCT^ method. All values were calculated from three independent biological replicates and presented as mean ± SD. *P<0.05, **P<0.01, for comparison between two indicated groups.** (B)** Schematic diagram of *Sufu* conditional allele (*Sufu^flox^*) which was created by flanking Exon 7 of the mouse *Sufu* gene with two parallel *LoxP* sites by gene targeting. **(C)** Schematic diagram* of LysM-Cre* knockin allele, in which Cre recombinase was under the control of the endogenous M lysozyme locus. **(D)** Schematic illustration of the mating strategy to obtain *Sufu^CKO^* (*LysM-Cre^+/+^; Sufu^flox/flox^*) mice and their littermate control mice (*LysM-Cre^+/+^*). **(E)** Schematic illustration of the strategy to disrupt *Sufu* gene in osteoclast precursors.** (F)** qPCR analyses of *Sufu* mRNA expression in macrophage-abundant spleens in *Sufu^CKO^* mice relative to their littermate controls (Ctrl). **(G-I)** qPCR analyses of mRNA expression of *Sufu* **(G)**,* Gli1* **(H)**, and *Ptch1* **(I)** in BMMs from *Sufu^CKO^* mice relative to their littermate controls (Ctrl). n=3 per group. All bar graphs were presented as mean ± SD, **P<0.01, compared with control group.

Sufu is a key intracellular suppressor of Hh signaling, and its inactivation was known to result in constitutively activated Hh signaling 45, 46. To explore the potential role of Sufu in regulating osteoclast differentiation, we first performed qPCR to examine the mRNA expression of *Sufu* gene during RANKL-induced osteoclastogenesis. The results showed that *Sufu* was abundantly expressed in BMMs and its mRNA level was modestly but statistically significantly increased with the progression of osteoclast differentiation (**Figure 1A**), implying that Hh signaling needed to be repressed by Sufu for osteoclast differentiation to proceed. To genetically assess the direct role of Hh activation in osteoclastogenesis, we next crossed mice carrying a conditional allele of *Sufu* (*Sufu^flox^*) to *LysM-Cre* mice to generate *LysM-Cre^+/+^; Sufu^flox/flox^* (hereafter *Sufu^CKO^*) mice (**Figure 1B-D**) in which *Sufu* will be specifically deleted in monocyte/macrophage lineage cells, including osteoclasts and their precursors (**Figure 1E**). qPCR analysis of RNA directly isolated from macrophage-abundant spleens showed significantly reduced expression of *Sufu* gene in *Sufu^CKO^* mice compared with their littermate controls (**Figure 1F**). To further verify the efficiency of *LysM-Cre*-mediated deletion of *Sufu* gene in macrophages, we isolated bone marrow macrophages (BMMs) from *Sufu^CKO^* and Ctrl (*LysM-Cre^+/+^*) mice, and cultivated them in complete culture medium containing 30 ng/ml M-CSF for 2 days prior to RNA isolation. qPCR experiment showed that the mRNA level of *Sufu in* BMM isolated from *Sufu^CKO^* mice was reduced by 82% when compared with that of control mice (**Figure 1G**). In contrast, the mRNA expression of* Gli1* and *Ptch1,* two well-established target genes of Hh signaling pathway, was elevated to 840.3 ± 106.9% and 175.3 ± 13.9%, respectively (**Figure 1H, I**). Taken together, our data demonstrated that *Sufu* gene was efficiently deleted in the macrophages of *Sufu^CKO^* mice, and that *Sufu* ablation in these cells resulted in activation of Hh signaling pathway.

### Genetic activation of Hh signaling by *Sufu* ablation inhibited RANKL-induced osteoclastogenesis and F-actin ring formation

**Figure 2:**
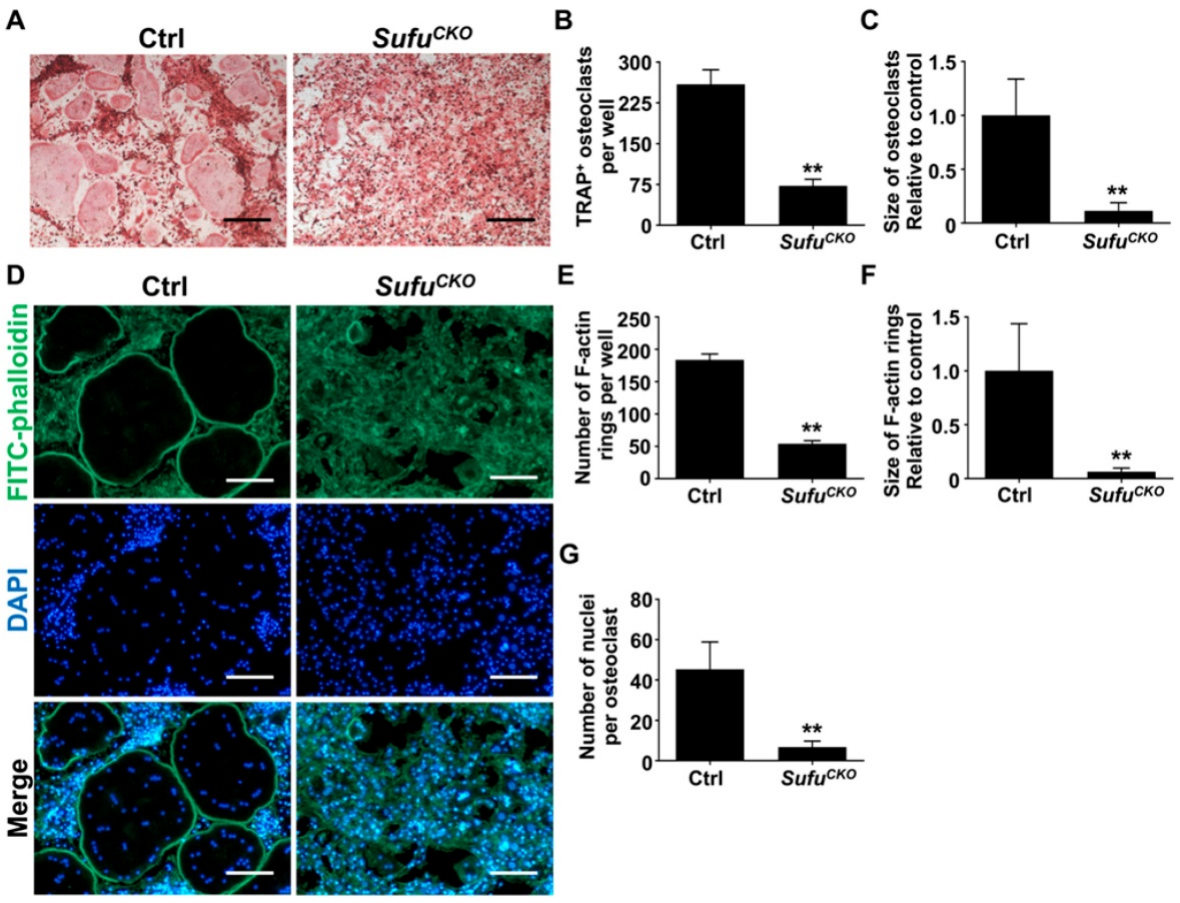
** Genetic activation of Hh signaling by *Sufu* ablation inhibited RANKL-induced osteoclastogenesis and F-actin ring formation *in vitro*. (A)** BMMs isolated from control and *Sufu^CKO^* mice were stimulated with 30 ng/ml M-CSF and 50 ng/ml RANKL for 6 days and then subjected to TRAP staining. Images shown were representative results from three independent experiments. Scale bar, 400 μm. **(B)** Quantification of number of TRAP^+^ multinucleated osteoclasts/well. **(C)** Quantification of relative size of TRAP^+^ multinucleated osteoclasts. Values were normalized to average size of TRAP^+^ osteoclasts in control group. **(D)** Representative images of F-actin rings. After stimulation with M-CSF and RANKL for 6 days, control and *Sufu^CKO^* BMM cultures were fixed with 4% PFA and then sequentially stained with FITC-phalloidin and DAPI. Green, F-actin; Blue, nuclei. Scale bar, 200 μm. **(E-G)** Quantitative analyses of F-actin rings showing number of F-actin rings per well (E), relative size of F-actin rings (F), and number of nuclei per osteoclast (G). All values were calculated from three independent replicates, and presented as mean ± SD. ** indicated *P*<0.01, relative to control group.

After confirming activation of Hh signaling following *Sufu* ablation, we next investigated the effects of *Sufu* ablation on osteoclast formation. To this end, BMMs were isolated from* Sufu^CKO^* and control mice, and subjected to osteoclastic induction in the presence of 50 ng/ml RANKL. As expected, abundant TRAP-positive multinucleated cells, indicative of mature osteoclasts, appeared in the control cultures after 6 days of RANKL treatment (**Figure 2A**). In contrast, formation of TRAP-positive osteoclasts was severely inhibited in the RANKL-treated *Sufu^CKO^* cultures (**Figure 2A**). Quantitative analyses showed that an average of 259 ± 15.4/well TRAP-positive osteoclasts was formed in the control cultures, whereas this number dropped to 72.3 ± 7.1/well in the *Sufu^CKO^* cultures (**Figure 2B**). Similarly, the size of TRAP-positive osteoclasts was significantly decreased in the control cultures compared with that in the *Sufu^CKO^* cultures (**Figure 2C**). These results clearly indicated that activation of Hh signaling by *Sufu* ablation directly suppressed osteoclastogenesis *in vitro*.

F-actin ring is a characteristic feature of mature osteoclasts, and required for their bone-resorbing activity 7, 38. To further validate that *Sufu* ablation impaired osteoclastogenesis, we analyzed F-actin ring formation in RANKL-treated control and *Sufu^CKO^* BMM cultures. Consistent with results from TRAP staining, phalloidin staining revealed that* Sufu^CKO^* BMMs formed significantly fewer number of F-actin rings than control BMMs after RANKL treatment (**Figure 2D, E**). Moreover, the average size of F-actin rings in multinucleated osteoclasts also significantly decreased by 93.3% in the *Sufu^CKO^* group when compared with that of the control group, and number of nuclei per osteoclast dropped from 45.3 ± 5.1 to 6.7 ± 1.1 (**Figure 2F, G**). Collectively, our data demonstrated that *Sufu* ablation inhibited RANKL-induced F-actin ring formation in BMMs, and therefore provided additional evidence to support the role of Hh activation in suppressing osteoclastogenesis.

### Genetic activation of Hh signaling by* Sufu* ablation suppressed expression of osteoclast-specific genes *in vitro*

**Figure 3:**
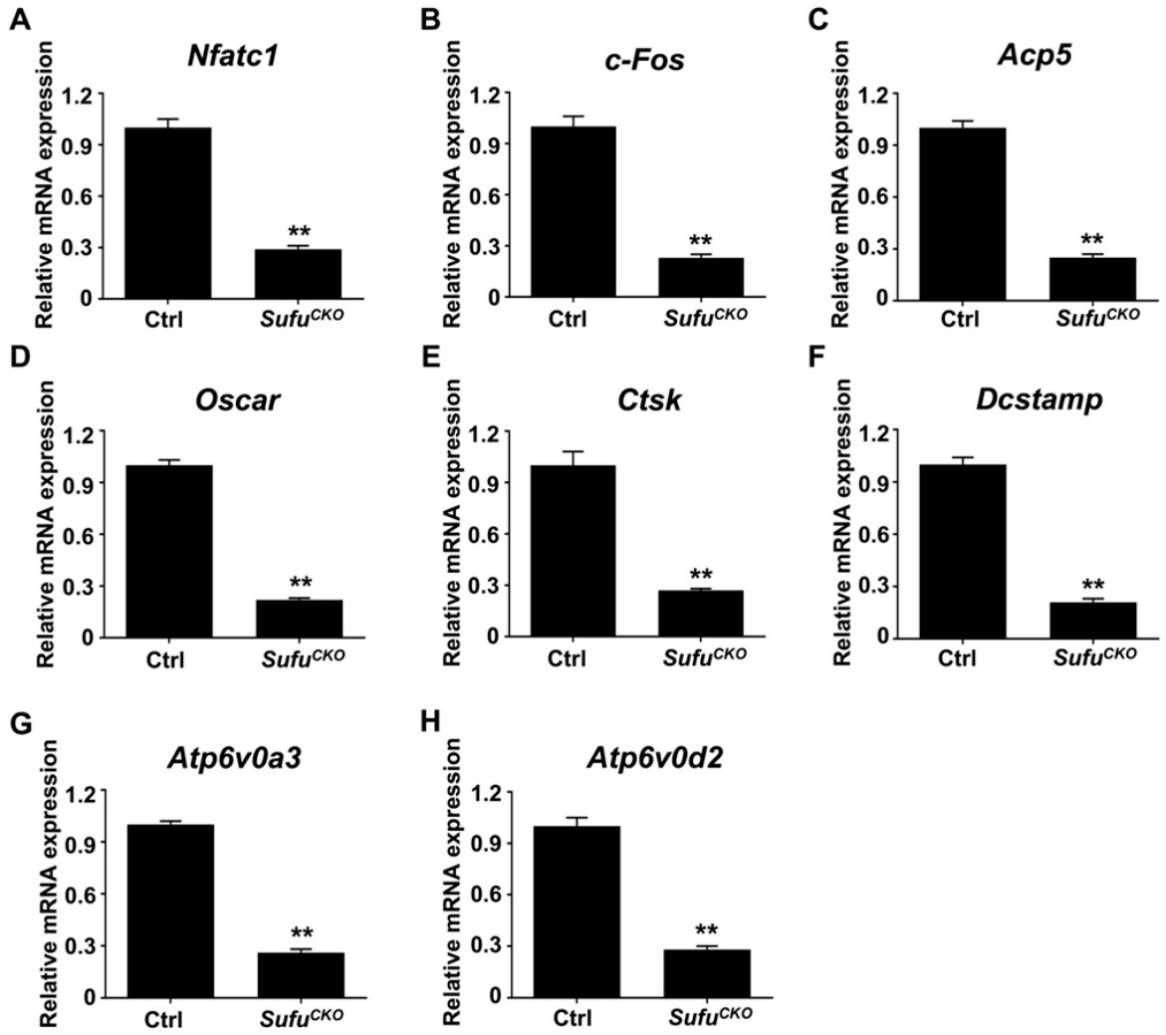
** Genetic activation of Hh signaling by* Sufu* ablation suppressed expression of osteoclast-specific genes *in vitro.*** qPCR analysis of relative mRNA levels of osteoclast-specific genes, including *Nfatc1* (**A**)*, c-Fos* (**B**)*, Acp5* (**C**)*, Oscar* (**D**)*, Ctsk* (**E**)*, Dcstamp* (**F**)*, Atp6v0a3* (**G**), and* Atp6v0d2* (**H**), in BMMs isolated from control and *Sufu^CKO^* mice at 6 days after RANKL stimulation. Expression of each target gene was normalized by 18S ribosomal RNA. The relative changes in mRNA level were analyzed by 2^-ΔΔCT^ method. All values were calculated from three independent biological replicates and presented as mean ± SD. **P<0.01, compared with control group.

The expression of a group of osteoclast-associated genes, including *Nfatc1, c-Fos, Acp5, Ctsk, Oscar, Dcstamp, Atp6v0a3* and* Atp6v0d2,* was upregulated during osteoclast differentiation 39, 47, 48*.* To further verify the inhibitory effect of *Sufu* ablation on osteoclast differentiation, we utilized quantitative RT-PCR to assess mRNA expression of these osteoclast differentiation markers in control and *Sufu^CKO^* BMMs that were induced toward osteoclasts with 6 days of RANKL stimulation. As shown in **Figure 3A&B**, mRNA levels of *Nfatc1 and c-Fos,* two key transcriptional regulators of osteoclast differentiation, significantly decreased to 29.3 ± 1.2% and 22.7 ± 1.2%, respectively, in the RANKL-stimulated *Sufu^CKO^* BMMs relative to control cells. Similarly, *Sufu* ablation dramatically suppressed expression of *Acp5, Oscar, Ctsk, Dcstamp, Atp6v0a3* and *Atp6v0d2* (**Figure 3C-H**), osteoclast marker genes that are known to be involved in various stages of osteoclast differentiation or their bone-resorbing activity*.* Collectively, these results demonstrated that activation of Hh signaling by *Sufu* ablation suppressed expression of osteoclast-specific genes, and thus osteoclast differentiation *in vitro.*

### Genetic activation of Hh signaling by *LysM-Cre*-mediated *Sufu* deletion suppressed osteoclastogenesis and prevented Ti particle-induced bone loss* in vivo*

**Figure 4:**
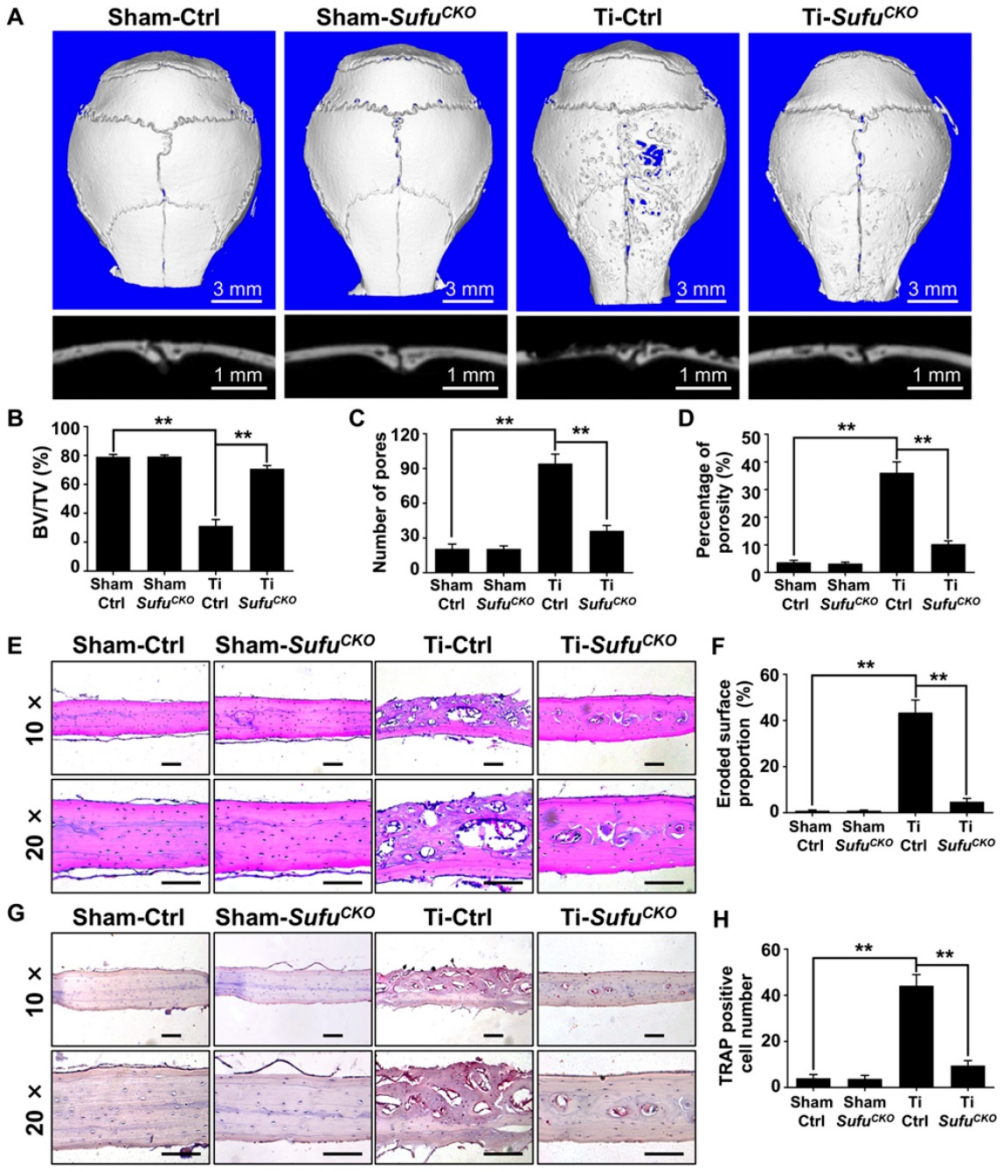
** Genetic activation of Hh signaling by *LysM-Cre*-mediated deletion of *Sufu* gene prevented Ti particle-induced bone loss and suppressed osteoclastogenesis* in vivo.* (A)** Representative 2D (bottom panels) and 3D (top panels) μCT images of calvariae from sham-operated control mice (Sham-Ctrl), *Sufu^CKO^* mice with sham operation (Sham-*Sufu^CKO^*), Ti particle-implanted control mice (Ti-Ctrl), and Ti particle-implanted *Sufu^CKO^* mice (Ti-*Sufu^CKO^*). **(B-D)** Quantitative μCT analyses of bone architecture parameters including bone volume/total tissue volume (BV/TV) **(B)**, number of pores **(C)** and percentage of porosity **(D)**. **(E)** Representative images of H&E staining of calvarial sections from Sham-Ctrl, Sham-*Sufu^CKO^*, Ti-Ctrl, and Ti-*Sufu^CKO^* mice. Scale bar, 100 μm. **(F)** Quantification of eroded surface proportion on H&E-stained calvarial sections. **(G)** Representative images of TRAP staining of calvarial sections from Sham-Ctrl, Sham-*Sufu^CKO^*, Ti-Ctrl, and Ti-*Sufu^CKO^* mice. Scale bar, 100 μm. **(H)** Quantitative analyses of number of TRAP-positive multinucleated cells. All bar graphs were presented as mean ± SD. n=5 mice per group, **P<0.01, determined by statistical analyses between two indicated groups. Sham-Ctrl, sham-operated control mice; Sham-*Sufu^CKO^, Sufu^CKO^* mice with sham operation; Ti-Ctrl, Ti particle-implanted control mice; Ti-*Sufu^CKO^*, Ti particle-implanted *Sufu^CKO^* mice.

Since genetic ablation of *Sufu* inhibited osteoclast formation *in vitro*, we next explored whether it could exert protective effects on wear particle-induced periprosthetic osteolysis, which is known to be primarily caused by excessive osteoclast formation and activity. To this end, we employed a murine calvarial model of Ti particle-induced osteolysis. Specifically, we first baked Ti particles for 6 h at 180 °C to remove endotoxins, and then confirmed that the baking treatment did not overtly alter the morphology and phase composition of Ti particles using SEM and XRD analyses, respectively (**Figure S1**). We implanted Ti particles on the calvariae of *Sufu^CKO^* and their littermate control mice, and analyzed calvarial bones two weeks after implantation. As revealed by 2D and 3D μCT imaging, Ti particles induced profound bone loss on the calvariae of the control mice, which was largely prevented in the *Sufu^CKO^*mice (**Figure 4A**). Quantitative μCT analyses further revealed that bone volume/total tissue volume (BV/TV) was markedly reduced, whereas both number of pores and the percentage of porosity were notably increased, in Ti particle-treated control mice when compared with those in sham-operated control mice (**Figure 4B-D**). Of note, these Ti particle-induced changes were significantly diminished in the *Sufu^CKO^*mice (**Figure 4B-D**). Taken together, these results suggested that *Sufu* ablation exerted protective effects on Ti particle-induced osteolysis.

To confirm the above μCT results, we next performed histological analyses on calvarial sections. H&E staining showed that extensive bone destruction occurred in Ti particle-treated control mice, but was rarely detected in sham-operated mice (**Figure 4E**). More importantly, such osteolytic changes were obviously reduced upon *Sufu* deletion (**Figure 4E**). Consistent with these morphological observations, histomorphometric analysis revealed that the percentage of eroded surface caused by Ti particles was significantly decreased in *Sufu^CKO^* mice, when compared with that of control mice (**Figure 4F**). Similarly, TRAP staining showed that Ti particles profoundly increased the number of osteoclasts, as evidenced by a large number of TRAP-positive osteoclasts lining eroded bone surfaces in Ti particle-stimulated mice (**Figure 4G**). However, significantly fewer osteoclasts were formed in* Sufu^CKO^* mice than in control mice in response to Ti particles (**Figure 4G, H**). Taken together, our data indicated that conditional deletion of *Sufu* in macrophage lineage cells can alleviate Ti particle-induced bone loss by suppressing osteoclastogenesis *in vivo*.

### Pharmacological activation of Hh signaling suppressed RANKL-induced osteoclastogenesis and osteoclast-specific gene expression without causing cytotoxicity

**Figure 5:**
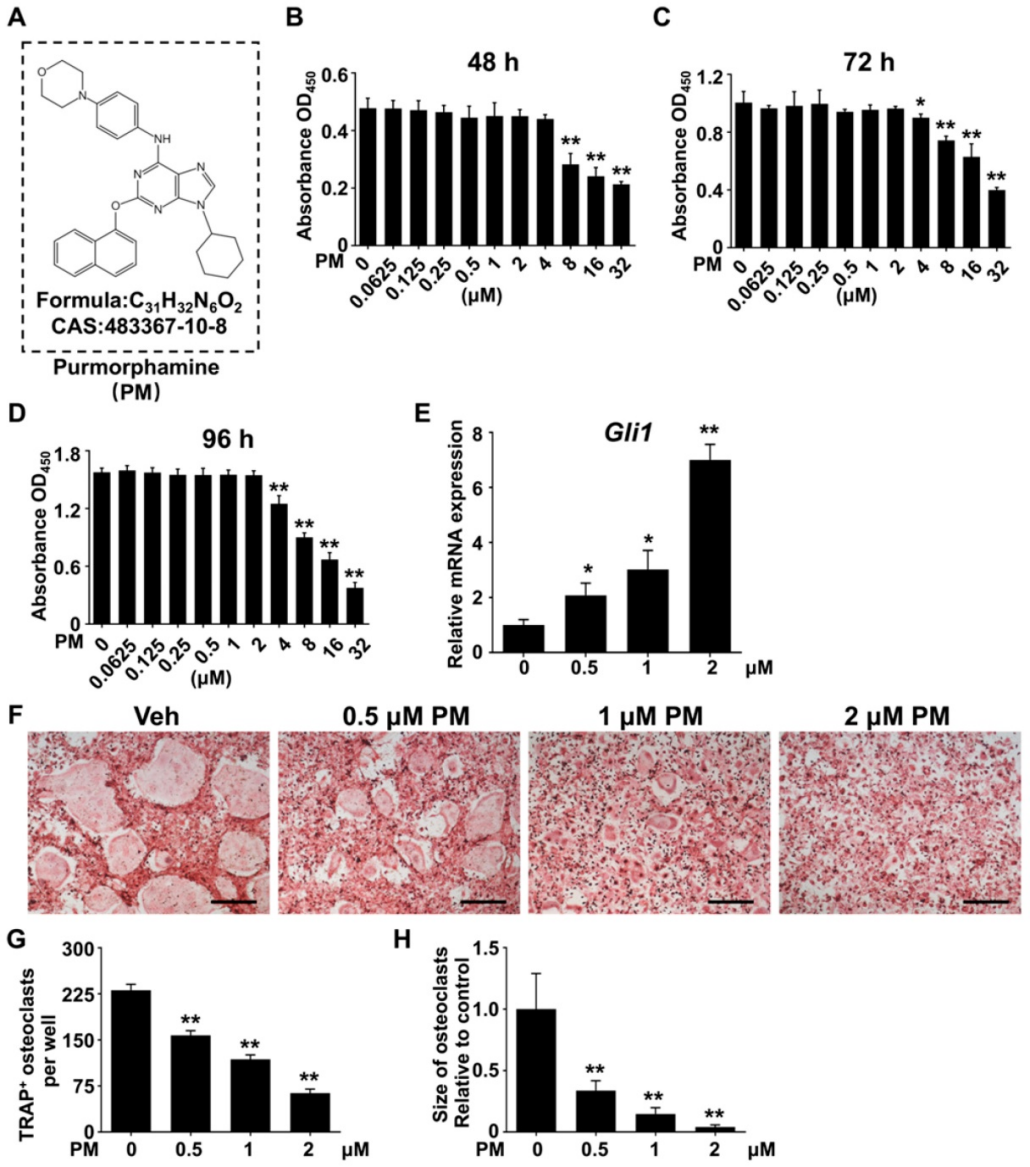
** Pharmacological activation of Hh signaling by PM suppressed RANKL-induced osteoclast formation without causing cytotoxicity *in vitro*. (A)** The molecular structure, formula and CAS number of PM. **(B-D)** BMMs were cultured with M-CSF in the presence of indicated concentrations of PM for 48 (B), 72 (C), or 96 h (D), then cell viability was measured by Cell Counting Kit-8 (CCK-8) assays. **(E)** qPCR analysis of relative *Gli1* mRNA expression in BMMs treated with 0, 0,5, 1, or 2 μM PM for 48 h. **(F)** BMMs were stimulated with 30 ng/ml M-CSF and 50 ng/ml RANKL for 6 days and then subjected to TRAP staining. Images shown were representative results from three independent replicates. Scale bar, 400 μm. **(G)** Quantification of number of TRAP^+^ multinucleated osteoclasts per well. **(H)** Quantification of relative size of TRAP^+^ multinucleated osteoclasts. All values were calculated from three independent biological replicates and presented as mean ± SD. *P<0.05, **P<0.01, relative to vehicle-treated group.

The above *in vitro* and *in vivo* genetic studies have established a protective role of Hh activation in Ti particle-induced osteolysis. These results inspired us to further test therapeutic efficacy of pharmacological activation of Hh signaling against osteolytic bone loss. To this end, we utilized purmorphamine (PM), a pharmacological activator of Smoothened (Smo) that is known to potently stimulate Hh signaling in a variety of cell types 19, 49-51. The molecular structure, formula, and CAS number of PM were shown in **Figure 5A**. To determine the potential cytotoxicity of PM, we performed CCK-8 assays to assess the viability of BMMs after being treated with different concentrations of PM for 48, 72, or 96 h. The results showed that PM at concentrations up to 2 μM did not exert any discernible effect on the viability of BMMs at all time points examined (**Figure 5B-D**). To confirm the stimulatory effect of PM on Hh signaling, we treated BMMs with PM at non-toxic concentrations (0, 0.5, 1 or 2 μM) for 2 days, and then examined their mRNA expression of *Gli1*, a direct Hh target gene commonly used as a readout of signaling activity. Indeed, qPCR analyses showed that PM treatments dose-dependently induced the transcription of *Gli1* in BMMs, therefore confirming activation of Hh signaling by PM in these cells (**Figure 5E**).

We next investigated the effect of Hh activation by PM on osteoclastogenesis. Osteoclastic differentiation of BMMs were induced with 50 ng/ml RANKL in the presence of vehicle (DMSO) or increasing concentrations of PM (0.5, 1 or 2 μM PM). After 6 days of induction, many large TRAP-positive multinucleated mature osteoclasts formed in the vehicle group (**Figure 5F**), whereas PM-treated groups were found to exhibit dose-dependent decreases in both number and size of these cells (**Figure 5F**). Quantitative measurement of TRAP^+^ osteoclasts further confirmed the above observations. While an average of 230.7 ± 5.6 osteoclasts were induced by RANKL stimulation in control BMM cultures, this number decreased to 157.3 ± 4.4, 118 ± 4.1, and 63.3 ± 3.7 in cells treated with 0.5, 1 and 2 μM PM, respectively (**Figure 5G**). Similarly, the average size of osteoclasts was also markedly diminished in PM-treated groups to 33.7 ± 2.1% at 0.5 μM, 14.6 ± 1.3% at 1 μM, and 4.1 ± 0.4% at 2 μM, relative to that of the vehicle-treated group (**Figure 5H**).

**Figure 6:**
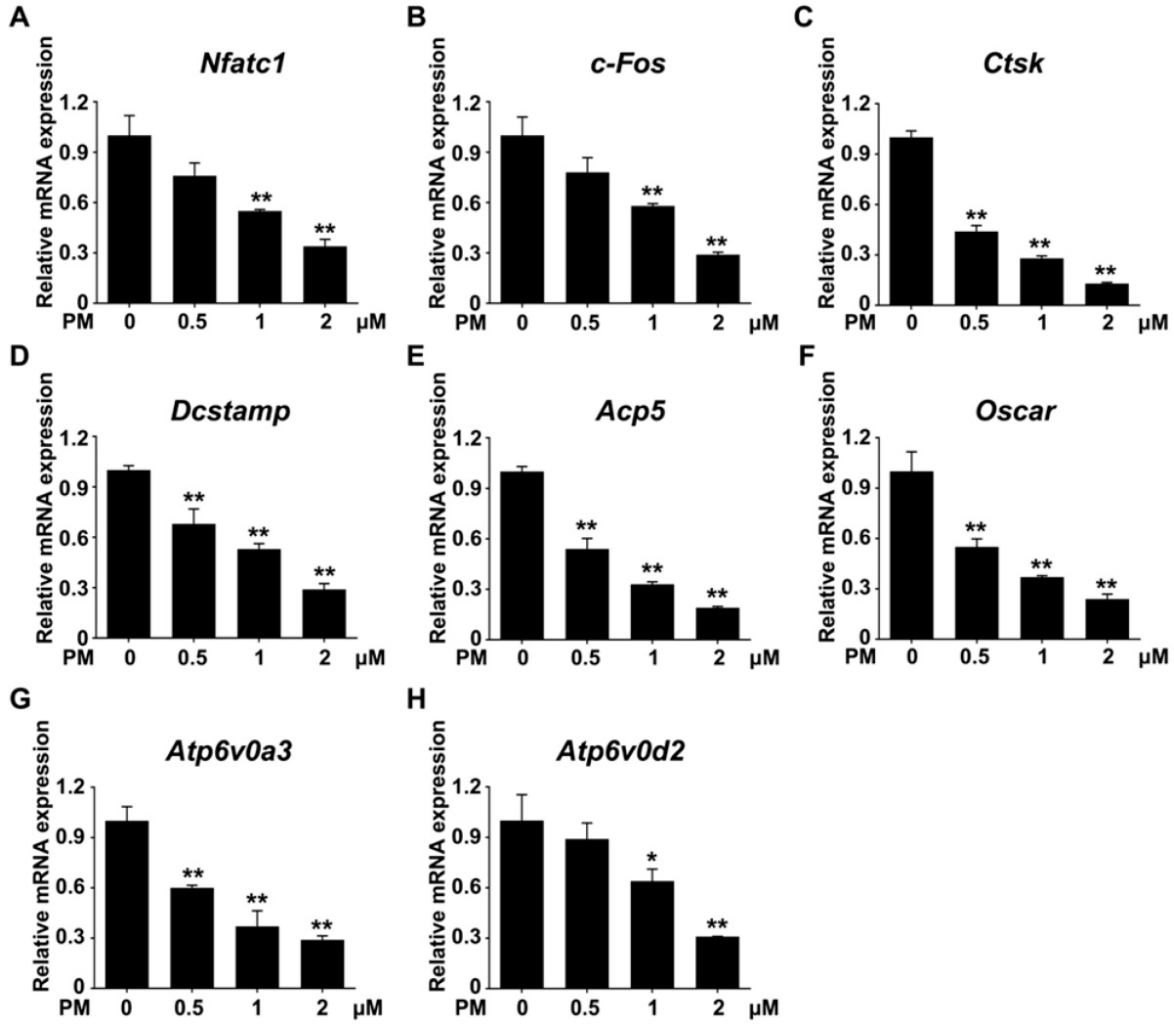
** Pharmacological activation of Hh signaling by PM restrained osteoclast-specific genes expressions under RANKL stimulation *in vitro*.** qPCR analysis of relative mRNA levels of osteoclast-specific genes, including *Nfatc1* **(A)***, c-Fos* **(B)***, Ctsk* **(C)***, Dcstamp* **(D)***, Acp5* **(E)***, Oscar* **(F)***, Atp6v0a3* **(G)**, and* Atp6v0d2* **(H)**, in BMMs stimulated with RANKL for 6 days in the presence of 0, 0.5, 1, or 2 μM PM. Expression of each target gene was normalized by 18S ribosomal RNA. The relative changes in mRNA level were analyzed by 2^-ΔΔCT^ method. All values were calculated from three independent biological replicates and presented as mean ± SD. **P<0.01, compared with vehicle-treated group.

Furthermore, the transcription levels of genes associated with osteoclast differentiation, including *Nfatc1, c-Fos, Acp5, Oscar, Ctsk, Dcstamp, Atp6v0a3,* and *Atp6v0d2*, were all markedly downregulated by PM treatment in a dose-dependent fashion (**Figure 6**). Thus, pharmacological activation of Hh signaling by PM can dose-dependently suppress RANKL-induced osteoclastogenesis and osteoclast-specific gene expression without causing cytotoxicity.

### Pharmacological activation of Hh signaling by PM inhibited RANKL-induced F-actin ring formation and osteoclastic bone resorption

**Figure 7:**
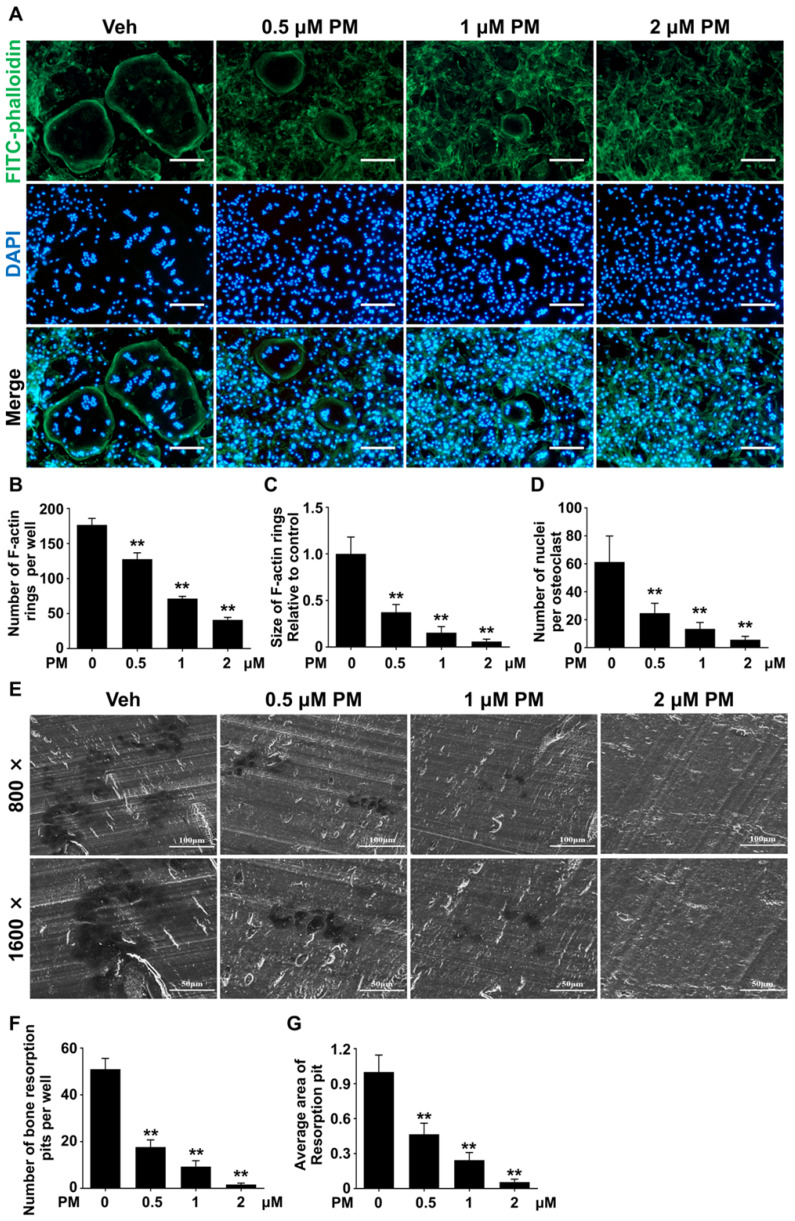
** Pharmacological activation of Hh signaling by PM inhibited RANKL-induced F-actin ring formation and osteoclastic bone resorption *in vitro*. (A)** Representative images of phalloidin staining of BMM clutures treated with RANKL for 6 days in the presence of 0, 0.5, 1, or 2 μM PM. F-actin rings. Green, F-actin; Blue, nuclei. Scale bar, 200 μm. **(B-D)** Quantitative analyses of F-actin rings showing number of F-actin rings per well **(B)**, relative size of F-actin rings **(C)**, and number of nuclei per osteoclast **(D)** in BMM clutures treated with RANKL for 6 days in the presence of 0, 0.5, 1, or 2 μM PM. **(E)** Representative scanning electron microscope (SEM) images of resorption pits on bovine slices seeded with differentiating osteoclasts in the presence of 0, 0.5, 1, or 2 μM PM. **(F-G)** Quantification of number (F) and average area (G) of bone resorption pits on SEM images. All values were calculated from three independent replicates, and presented as mean ± SD. ** indicated *P*<0.01, compared with vehicle-treated group.

To further confirm the effect of PM on osteoclastogenesis, we assessed RANKL-induced osteoclast F-actin ring formation. RANKL-treated BMMs were stained with phalloidin and DAPI to visualize F-actin ring and nuclei, respectively. The results showed that RANKL effectively induced formation of large F-actin ring in multinucleated cells, which was notably suppressed by PM supplementation (**Figure 7A**). Compared with control group, PM-treated groups exhibited significant reductions in both number and size of F-actin rings in a dose-dependent manner (**Figure 7B-D**). Of note, in cells treated with 2 μM PM, formation of F-actin rings was nearly completely disrupted (**Figure 7A**). Collectively, these findings suggested that pharmacological activation of Hh signaling by PM impaired osteoclast F-actin ring formation *in vitro*.

Since PM severely impaired osteoclastogenesis and F-actin ring formation, we predicted that osteoclastic bone resorption would also be inhibited by PM. To test this hypothesis, we performed an osteoclastic bone resorption assay. In this assay, differentiating BMMs were cultured on bovine bone slices, and then further induced to differentiate into mature osteoclast with RANKL stimulation. The resorption pits created by these osteoclasts were then analyzed by scanning electron microscopy (SEM). As shown in **Figure 7E**, several large resorption pits clearly formed in the vehicle group whereas fewer and smaller resorption pits were observed in PM groups. In line with impaired osteoclast formation, the number of resorption pits decreased significantly upon PM treatment, from 51 ± 2.7 in vehicle-treated group to 17.7 ± 1.8, 9.3 ± 1.5, and 1.7 ± 0.3 in groups treated with 0.5 μM, 1 μM, and 2 μM PM, respectively.

Furthermore, groups treated with 0.5, 1, and 2 μM PM exhibited 53.3 ± 3.9%, 75.6 ± 2.7%, and 94.4 ± 1.0% decreases in the average area of per bone resorption pit, respectively, when compared to that of vehicle-treated cells (**Figure 7F, G**). Taken together, these results demonstrated that activation of Hh signaling by PM treatment not only impaired osteoclastogeneis, but also inhibits osteoclast bone resorption activity *in vitro*.

### Pharmacological activation of Hh signaling by PM attenuated Ti particle-induced osteolysis *in vivo*

**Figure 8:**
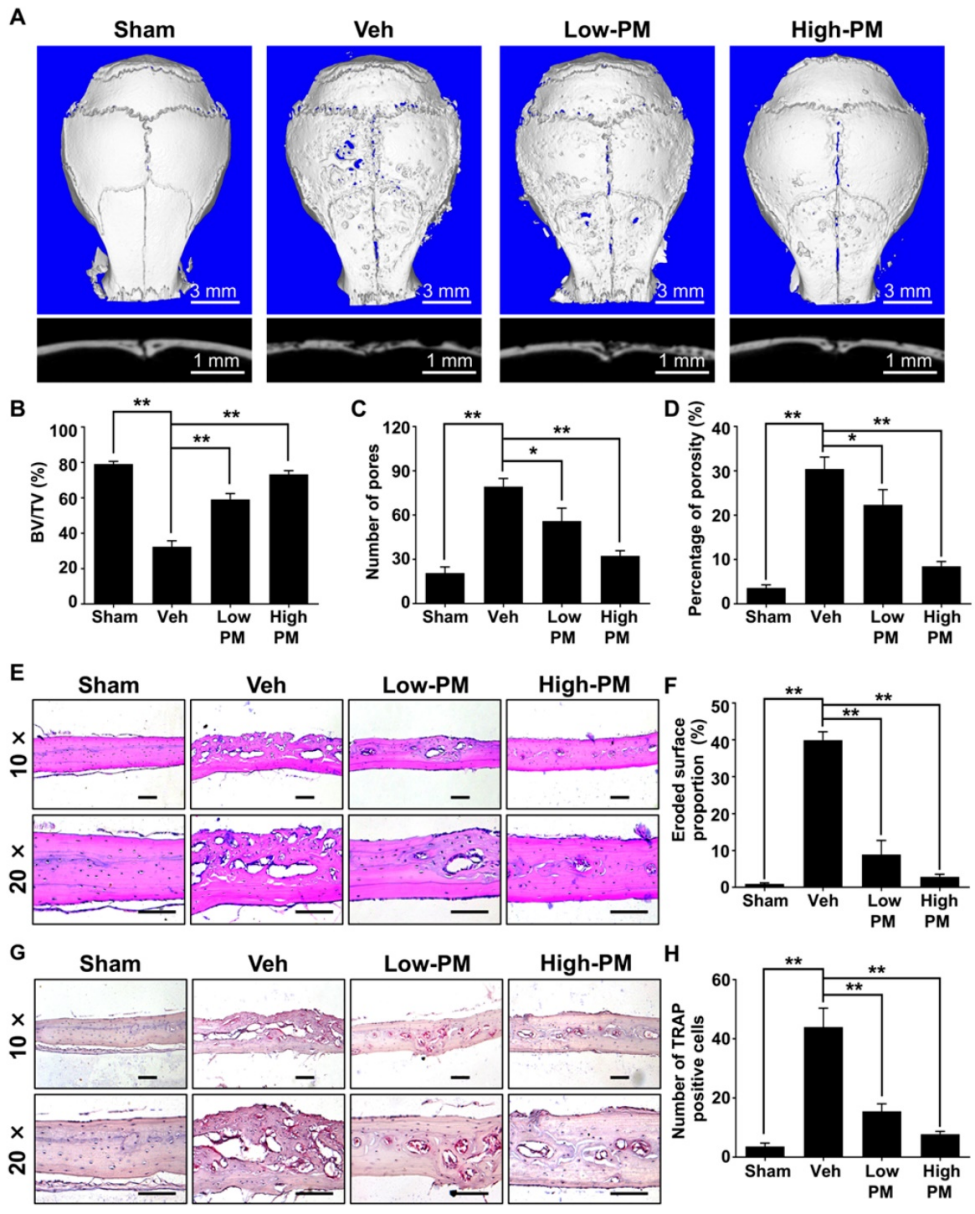
** Pharmacological activation of Hh signaling by PM attenuated Ti particle-induced osteolysis *in vivo*. (A)** Representative 2D (bottom panels) and 3D (top panels) μCT images of calvariae from sham-operated mice (Sham) and Ti particle-implanted mice subjected to administration with vehicle (Veh), 2.5 mg/kg PM (Low-PM), or 10 mg/kg PM (High-PM). **(B-D)** Quantitative μCT analyses of bone architecture parameters including bone volume/total tissue volume (BV/TV) **(B)**, number of pores **(C)** and percentage of porosity **(D)**. **(E)** Representative images of H&E staining of calvarial sections from sham-operated mice (Sham) and Ti particle-implanted mice subjected to administration with vehicle (Veh), 2.5 mg/kg PM (Low-PM), or 10 mg/kg PM (High-PM). Scale bar, 100 μm. **(F)** Quantification of eroded surface proportion on H&E-stained calvarial sections. **(G)** Representative images of TRAP staining of calvarial sections from sham-operated mice (Sham) and Ti particle-implanted mice subjected to administration with vehicle (Veh), 2.5 mg/kg PM (Low-PM), or 10 mg/kg PM (High-PM). Scale bar, 100 μm.** (H)** Quantitative analyses of number of TRAP-positive multinucleated cells. All bar graphs were presented as mean ± SD. n=7 mice per group, *P<0.05, **P<0.01, determined by statistical analyses between two indicated groups.

Having established that pharmacological activation of Hh signaling by PM suppressed RANKL-induced osteoclastogenesis in *vitro*, we then investigated whether PM could protect against particle-induced bone loss *in vivo*. Mice were implanted with Ti particles on the calvariae before being subjected to daily injections of vehicle, low or high dosages of PM (2.5 mg/kg and 10 mg/kg, respectively) for 2 weeks. As shown in **Figure 8A**, Ti particles induced massive bone loss in vehicle-treated calvariae, which was clearly suppressed by treatment of PM in a dose-dependent manner. Consistently, quantification of μCT images further revealed that BV/TV of the vehicle group was significantly decreased compared with that of the sham group (32.3 ± 1.9% vs. 79.1 ± 0.9%), whereas such reduction in bone mass is considerably reversed in low- and high-PM groups (59 ± 1.9% and 73.3 ± 1.2%, respectively) (**Figure 8B**). Similarly, PM treatments dose-dependently decreased the number of pores as well as the percentage of porosity in Ti particle-treated calvariae (**Figure 8C, D**).

To further verify the therapeutic effect of PM on Ti particle-induced osteolysis *in vivo*, we performed histological and histomorphometric analysis on calvarial sections. H&E staining revealed that Ti particles caused severe bone erosion in the calvariae of vehicle group, which was significantly attenuated in low-PM group, and almost completely blocked in high-PM group (**Figure 8E**). Statistical analyses further revealed that PM markedly reduced the percentage of eroded bone surface out of total bone surface, an indicator reflecting degrees of bone destruction, from 38.9 ± 1.1% in the vehicle group to 8.9 ± 1.9% and 2.9 ± 0.4% in low- and high-PM groups, respectively (**Figure 8F**). Furthermore, TRAP staining showed that TRAP-positive multinucleated cells accumulated in the osteolytic regions predominantly along the eroded bone surfaces in Ti particle-implanted mice (**Figure 8G**). Importantly, the average numbers of osteoclasts in low- and high-PM groups reduced to 15.5 ± 1.3 and 7.8 ± 0.5, respectively, which were significantly less than 44 ± 3.2 in the vehicle group (**Figure 8H**).

Taken together, our results demonstrated that pharmacological activation of Hh signaling by PM protected against Ti particle-induced osteolysis in a dose-dependent manner.

### Hh signaling negatively regulates the JNK/c-Fos-NFATc1 cascade during osteoclast differentiation

**Figure 9:**
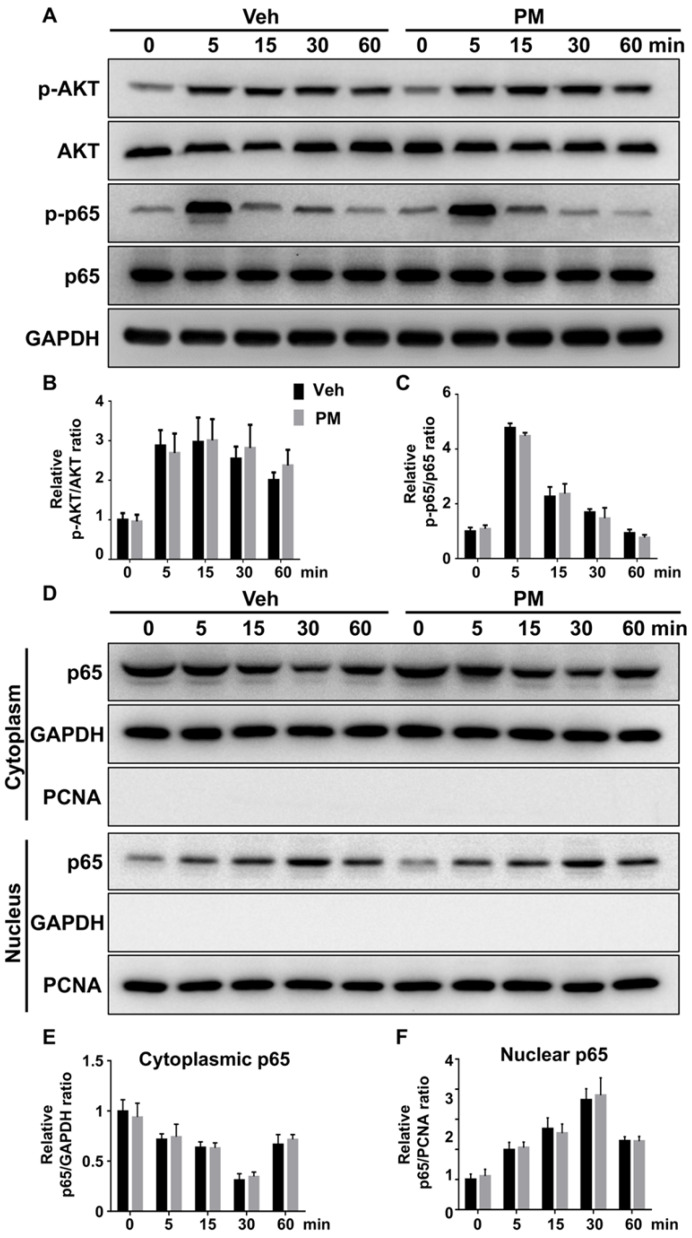
** Activation of Hh signaling did not affect RANKL-induced activation of AKT and NF-κB pathways. (A)** Western blot analyses of protein levels of phosphorylated AKT (p-AKT), total AKT, phosphorylated NF-κB p65 (p-p65), total NF-κB p65 (p65), and GAPDH in BMMs treated with 50 ng/ml RANKL for indicate times in the presence of vehicle (Veh) or 2 µM PM (PM). Representative images from three independent biological replicates were shown.** (B-C)** Quantitative analyses of relative ratios of p-AKT/AKT (B) and p-p65/p65 (C). n=3 per group.** (D)** Western blot analyses of protein levels of NF-κB p65 (p65) in the cytoplasmic and nuclear extracts from BMMs treated with 50 ng/ml RANKL in the presence of vehicle (Veh) or 2 µM PM (PM) for indicate times. Representative images from three independent biological replicates were shown. **(E-F)** Quantitative analyses of relative ratios of cytoplasmic p65/GAPDH (E), and nuclear p65/PCNA (F). n=3 per group. All bar graphs were presented as mean ± SD.

Multiple signaling pathways, including NF-κB, PI3K-Akt, and MAPKs, are activated by RANKL and involved in various aspects of osteoclastogenesis 48, 52-55. To explore the mechanism underlying the inhibitory effect of Hh signaling on osteoclast formation, we investigated the effects of PM on RANKL-induced activation of these pathways. BMMs were pretreated with vehicle or 2 µM PM for 4 h, then stimulated with 50 ng/ml RANKL for 0, 5, 15, 30 and 60 min before being subjected to qPCR and Western blot analyses. qPCR analysis revealed an increase in mRNA expression of *Gli1* in PM-treated BMMs compared to control cells, therefore verifying activation of Hh signaling in these cells (**Figure S2**). Western blot analysis further showed that RANKL potently increased levels of phosphorylated NF-κB p65 and AKT, which was not significantly affected by PM treatment at all time points examined (**Figure 9A-C**). In addition to phosphorylation of NF-κB p65, its nuclear translocation is a critical event for activation of NF-κB signaling 56. To determine whether PM affected the RANKL-induced nuclear translocation of p65 in BMMs, we further examined protein levels of p65 in the cytoplasmic and nuclear extracts from BMMs treated as described above. As shown in **Figure 9D-F**, RANKL-induced nuclear translocation of p65 was not significantly altered in PM-treated BMMs compared to vehicle-treated cells. Thus, the inhibitory effect of Hh signaling on osteoclast differentiation was unlikely mediated by NF-κB and AKT pathways.

**Figure 10:**
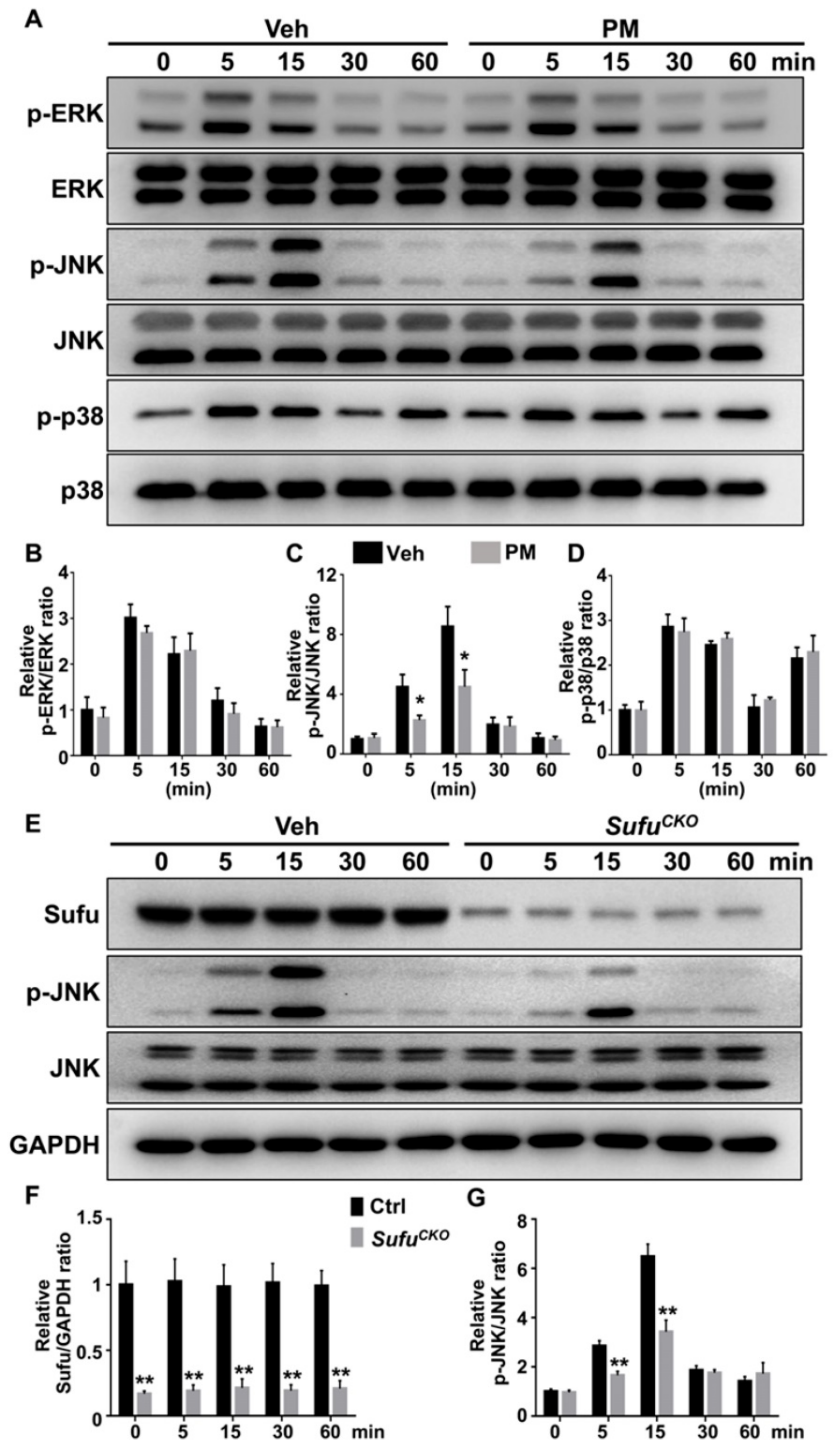
** Activation of Hh signaling inhibited RANKL-induced activation of JNK pathway. (A)** Western blot analyses of protein levels of phosphorylated ERK (p-ERK), total ERK, phosphorylated JNK (p-JNK), total JNK, phosphorylated p38 (p-p38), and total p38 in BMMs treated with 50 ng/ml RANKL in the presence of vehicle (Veh) or 2 µM PM (PM) for indicate times. Representative images from three independent biological replicates were shown. **(B-D)** Quantitative analyses of relative ratios of p-ERK/ERK (B), p-JNK/JNK(C) and p-p38/p38 (D). n=3 per group. All bar graphs were presented as mean ± SD. *P<0.05, compared with vehicle-treated (Veh) group. **(E)** Western blot analyses of protein levels of Sufu, phosphorylated JNK (p-JNK), total JNK, and GAPDH in BMMs from control and *Sufu^CKO^* (CKO) mice after treatment with 50 ng/ml RANKL for indicate times. Representative images from three independent biological replicates were shown. **(F-G)** Quantitative analyses of relative ratios of Sufu/GAPDH (F), and p-JNK/JNK(G). **P<0.01, compared with control groups.

For analyzing the MAPK pathway, we examined phosphorylation status of its family members including ERK, JNK and p38. After RANKL stimulation, levels of the phosphorylated ERK, JNK, and p38 were rapidly induced, and peaked at 5, 15, and 5 min, respectively (**Figure 10A-D**). Interestingly, PM notably decreased phosphorylation of JNK at 5 and 15 min after RANKL treatment (**Figure 10A-D**), whereas it did not significantly alter RANKL-induced phosphorylation of p38 kinase and ERK (**Figure 10A-D**). Similarly, *Sufu* deletion significantly impaired RANKL-induced phosphorylation of JNK (**Figure 10E-G**). Thus, Hh signaling appeared to exert a specific inhibitory effect on RANKL-induced activation of JNK signaling.

**Figure 11:**
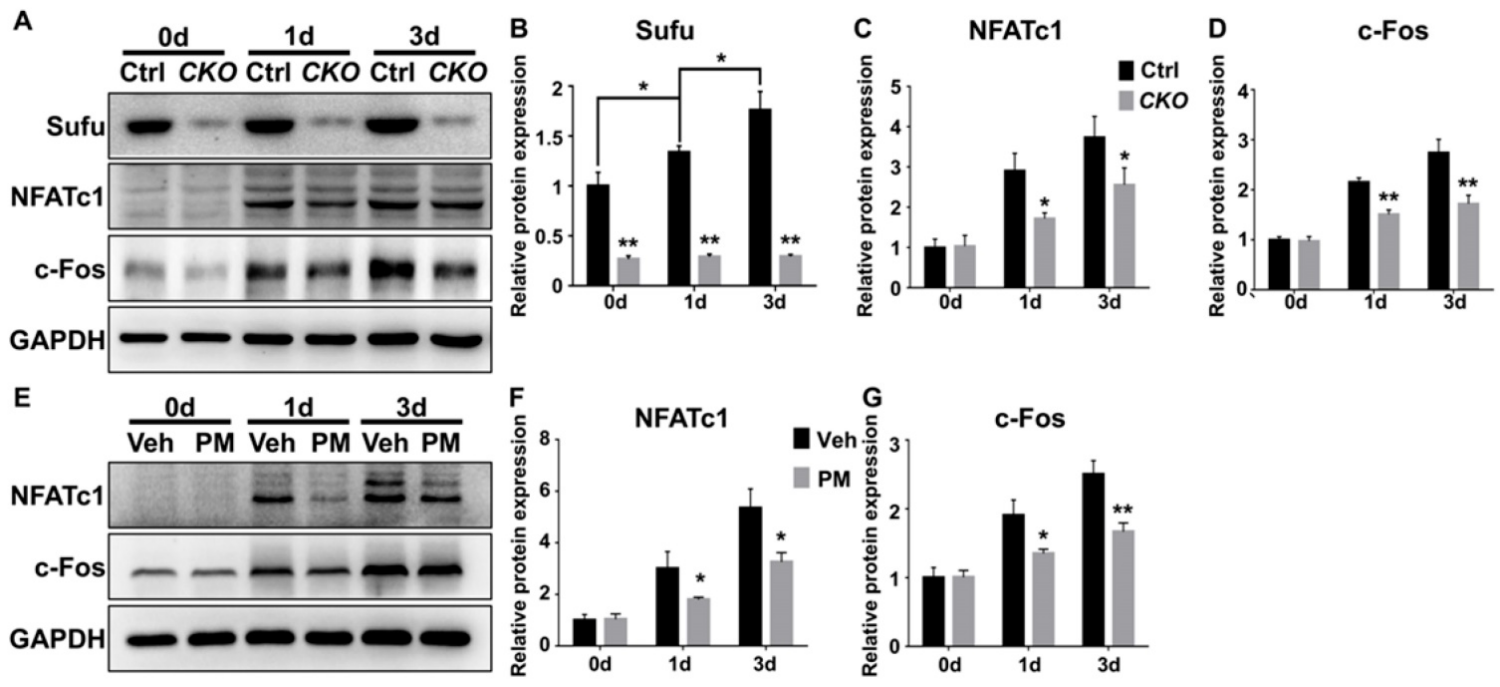
** Activation of Hh signaling suppressed NFATc1 and c-Fos expression during osteoclastogenesis. (A)** Western blot analyses of protein levels of Sufu, NFATc1 and c-Fos in BMMs from control and *Sufu^CKO^* (CKO) mice at 0, 1, and 3 days after RANKL stimulation. Images shown were representative results from three independent biological replicates.** (B-D)** Quantification of relative protein levels of Sufu (B), NFATc1 (C) and c-Fos (D). All values were normalized to protein level of GAPDH, and shown as mean ± S.D. n=3 per group. *P<0.05, **P<0.01, compared with control or indicated group.** (E)** Western blot analyses of protein levels of NFATc1 and c-Fos in vehicle- (Veh) or PM-treated (PM) BMMs at 0, 1, and 3 days after RANKL stimulation. Images shown were representative results from three independent biological replicates.** (F-G)** Quantification of relative protein levels of NFATc1(F) and c-Fos (G). GAPDH was used as a loading control. All bar graphs were presented as mean ± SD. n=3 per group. *P<0.05, **P<0.01, compared with vehicle-treated (Veh) group.

NFATc1 is a master transcriptional regulator of osteoclast differentiation that functions downstream of JNK signaling and c-Fos protein, another transcriptional regulator essential for osteoclast differentiation and function 52, 55, 57. JNK pathway phosphorylates and thus activates activity of c-Jun, which forms the activator protein 1 (AP-1) complex with c-Fos to induce and amplify *Nfatc1* expression 52, 55, 57, 58. Of note, the above qPCR analyses have shown that genetic or pharmacological activation of Hh signaling significantly down-regulated mRNA levels of both *Nfatc1* and *c-Fos* in RANKL-treated BMMs. To further confirm these results, we performed Western blot analyses to analyze the effect of Hh activation on RANKL-induced expression of NFATc1 and c-Fos proteins. As shown in **Figure 11**, levels of NFATc1 and c-Fos proteins were highly elevated in BMMs following 1 and 3 days of RANKL stimulation. However, such inductions were significantly reduced in BMMs upon *Sufu* deletion or PM treatment. Taken together, our results strongly suggested that Hh signaling cell-autonomously hindered osteoclastogenesis through suppressing JNK- and c-Fos-mediated regulation of NFATc1.

## Discussion

PPO is a common long-term complication of TJA operations, and represents the major cause of aseptic loosening and subsequent implant failure, which often leads to revision surgery and thus causes tremendous economic as well as psychological burdens on patients and their families. Since PPO is predominantly caused by hyperactive bone resorption activated by wear particles liberated from implant surfaces, current clinical treatments are mainly focused on inhibiting osteoclast formation and/or activity. However, several anti-resorption drugs, such as bisphosphonates and denosumab, exhibited limited therapeutic efficacy against this disease 9-11, not to mention that long-term use of these drugs could potentially cause severe side effects, such as osteonecrosis of the jaw and atypical femur fracture 59, 60. Moreover, these anti-resorption drugs cannot promote bone regeneration to repair pre-existing osteolytic lesions. Therefore, it is urgent to search for novel alternative agents or approaches that can better prevent and treat PPO. Here, we demonstrated that genetic or pharmacological activation of Hh signaling potently inhibited RANKL-induced osteoclastogenesis *in vitro* and strongly protected against Ti particle-induced bone loss in a murine calvarial model *in vivo*. Combined with the established bone anabolic role of Hh pathway, our findings suggested that activation of Hh signaling could be a promising therapeutic approach with both bone anabolic and anti-resorptive properties for battling PPO and other osteolytic diseases.

A few previous studies have explored the cell-autonomous effect of Hh pathway activation on osteoclast differentiation* in vitro*, but reported inconsistent results. Shimo et al. reported that Shh treatment notably enhanced RANKL-induced osteoclast differentiation of RAW264.7 cells, BMMs, and CD11b^+^ bone marrow cells 29. Similarly, Li et al. showed that pharmacological activation of Hh signaling promoted RANKL-induced formation of osteoclasts from RAW264.7 cells in the condition of estrogen deficiency 27. However, Heller et al. demonstrated that genetic activation of Hh signaling by removing one copy of *Ptch1* in BMMs did not affect the number of osteoclasts formed upon RANKL stimulation 28. In contrast, Tibullo et al. showed that Shh signaling mediated the inhibitory effect of Ixazomib (a third-generation proteasome inhibitor) on RANKL-induced osteoclastogenesis in human monocytes 30. Thus, these studies did not reach a consensus about the cell-autonomous role of Hh signaling in regulating osteoclastogenesis* in vitro*. Moreover, none of these studies evaluated the direct effects of enhanced Hh signaling on osteoclast formation *in vivo* under either a physiological or a pathological condition. Recently, a genetic study showed that specific ablation of *Ihh* in limb mesenchymal cells resulted in increased osteoclast formation, suggesting that Ihh signaling could play a physiological role in inhibiting osteoclastogenesis *in vivo* 17. However, ablation of *Ihh* in limb mesenchymal cells could potentially inactivate Ihh signaling in both osteoblastic and osteoclastic linage cells. Thus, it remains unclear whether Ihh signaling functions within osteoclast precursors to negatively regulate osteoclastogenesis. To clarify the cell-autonomous role of Hh signaling in osteoclastogenesis, we activated Hh signaling in primary BMMs by utilizing *LysM-Cre* to specifically ablate *Sufu*, a key negative regulator of Hh signaling in these cells or by directly treating them with PM, a pharmacological agonist of Smo. We then performed comprehensive analyses to evaluate direct effects of Hh activation on RANKL-induced osteoclastogenesis. We found that activation of Hh signaling by either approach suppressed osteoclast formation, F-actin ring formation, and osteoclast-specific gene expression. In addition, we observed the reduced number and area of bone resorption pits upon PM treatment in bone resorption assays. Taken together, our data have clearly demonstrated that enhancing Hh signaling cannot only directly restrain RANKL-induced formation of osteoclasts, but also impair their bone-resorbing capacity *in vitro.* Our results are seemingly at odds with the earlier findings described above. Although the reason for these conflicting results is still unclear, the following differences between our study and others may account for these discrepancies. First, we activated Hh signaling at the level of Smo or its downstream component Sufu, while studies by Shimo et al. and Heller et al. modulated this pathway by inhibiting Ptch1 activity or reducing its expression, respectively. It is possible that manipulation of Hh-Ptch1 signaling can exert a stimulatory impact on osteoclastogenesis via a Smo/Sufu-independent mechanism, while Smo/Sufu-mediated canonical Hh signaling acts to suppress bone resorption. Second, we used primary BMMs in the entire study, whereas others performed some experiments with RAW264.7 cells and CD11b^+^ bone marrow cells. Different types of osteoclast precursors may respond differently to RANKL induction upon Hh stimulation. Third, we used 50 ng/ml RANKL to induce osteoclast differentiation in the normal condition, while Li et al. performed osteoclastogenesis with 100 ng/ml RANKL in the condition of estrogen depletion. Hh signaling may play opposite roles in osteoclastogenesis under different conditions. While the discrepancies between our study and others surely need to be resolved, our studies have clearly demonstrated that *Sufu* deletion or PM treatment not only potently inhibited osteoclast differentiation *in vitro*, but also strongly suppressed Ti particle-induced osteoclastogenesis *in vivo*. Thus, enhanced Hh signaling can play a cell-autonomous role in inhibiting osteoclastogenesis.

The role of Hh signaling in regulating osteoclastogenesis is likely complicated. While our study provided solid evidence to support a cell-autonomous role of Hh signaling in suppressing osteoclast differentiation and activity, previous studies have demonstrated that Hh signaling can indirectly promote osteoclast differentiation by activating the PTHrP-PKA-CREB cascade in mature osteoblasts to upregulate their expression of RANKL, the potent inducer of osteoclastogenesis 22-24. Similarly, Hh ligands secreted by some tumor cells can stimulate osteoblast expression of RANKL to indirectly promote osteoclastogenesis and subsequent bone destruction 25, 26. Collectively, these studies indicated that Hh signaling can negatively and positively regulate osteoclastogenesis via cell-autonomous and non-cell autonomous mechanisms, respectively. Clearly, the ultimate effect of systemic Hh pathway activation on osteoclastogenesis is determined by the balance between these two distinct mechanisms, and probably varies depending on different physiological or pathological conditions. Therefore, it should be individually tested for each osteolytic disease whether Hh activation can be used to inhibit bone destruction. Using a murine calvarial model of Ti-particle-induced osteolysis, we showed that PM administration, which presumably activated Hh signaling in both osteoblasts and osteoclasts, led to suppression of Ti particle-induced osteoclast formation. Thus, the direct inhibitory effect of Hh signaling on osteoclastogenesis appeared to be dominant over its indirect stimulatory effect in this particular osteolytic condition.

Our results showed that inhibition of osteoclastogenesis by conditional ablation of *Sufu* in osteoclast precursors was sufficient to effectively alleviate Ti particle-induced bone loss, confirming the predominant role of osteoclasts in this osteolytic disease. In addition to enhanced bone resorption, recent studies indicated that impaired bone formation is also a contributing factor of this disease. These studies showed that wear particles cannot only directly impair osteogenesis and bone-forming capacity of osteoblasts 61, 62, but also indirectly suppress osteoblast differentiation and function through stimulating production of proinflammatory factors by macrophages 62, 63. Based on these facts, simultaneously suppressing bone resorption and promoting bone formation is likely a better strategy to prevent and treat PPO. In addition to its anti-resorption activity, our data suggested that activating Hh signaling in osteoclast precursors probably also exerted its therapeutic effect on Ti particle-induced bone loss through indirectly enhancing bone formation. Indeed, TRAP staining revealed that activation of Hh pathway in osteoclast precursors by either *Sufu* deletion or PM treatment markedly suppressed RANKL-induced formation of TRAP^+^ multinucleated osteoclasts, resulting in accumulation of TRAP^+^ mononuclear preosteoclasts. Preosteoclasts were known to secrete platelet-derived growth factor-BB (PDGF-BB) to stimulate formation of type-H vessels, which in turn promoted osteogenesis and bone formation 64, 65. Thus, accumulated preosteoclasts in *Sufu*-ablated or PM-treated mice could indirectly enhance bone formation through promoting type-H vessel formation.

Previous studies have shown that Hh signaling played a crucial role in osteoblastogenesis, and that forced activation of this pathway by both genetic or pharmacological approaches can potently stimulate osteogenic differentiation and bone formation *in vitro* and *in vivo* 14, 15, 20, 37, 49, 66-69. In line with these earlier findings, we also found that ablation of *Sufu* in BMSCs or treatment of these cells with PM caused accelerated osteoblast differentiation (data not shown). Thus, the protective effects of PM on Ti particle-induced bone loss observed in this study are probably in part attributed to its direct action on osteoblast differentiation and bone formation. In summary, our study and others collectively indicated that activation of Hh signaling cannot only suppress bone resorption, but may also promote bone formation through both indirect and direct mechanisms, therefore representing a promising therapeutic approach to prevent and treat PPO.

We should acknowledge that our study did have several limitations. Firstly, we used Ti particle-induced osteolysis as a model of PPO, despite the fact that the majority of wear particles present around metal-on-polyethylene implants are polyethylene particles 41, 70. However, titanium particles do represent one of the pathological factors eliciting osteolysis. Moreover, the pathogenesis of Ti particle- and polyethylene particle-induced osteolysis is similar, and both types of particles cause bone destruction predominantly through activating osteoclast formation and function 41, 70. In addition, Ti particle-induced osteolysis is the most commonly-used model for evaluating the effects of pharmaceuticals on wear particle-induced PPO, thus allowing comparison of our findings with others. Based on the above facts, we utilized this animal model in our study despite its potential limitation. It is no doubt that further studies are warranted to confirm our findings using osteolytic animal model that closely mimics the clinical features of PPO. Secondly, our study did not address whether targeting Hh pathway can treat pre-existing bone destruction since *Sufu* ablation and PM administration in this study were performed before osteolytic bone destruction has occurred. However, our findings did demonstrate that targeted activation of Hh signaling can inhibit osteoclastogenesis both *in vitro* and *in vivo*. Moreover, previous studies have established an anabolic role of Hh signaling in bone formation. Based on these findings, we predicted that activation of Hh signaling could not only prevent but also treat wear particle-induced PPO. Clearly, additional experiments should be performed to further verify this prediction.

**Figure 12:**
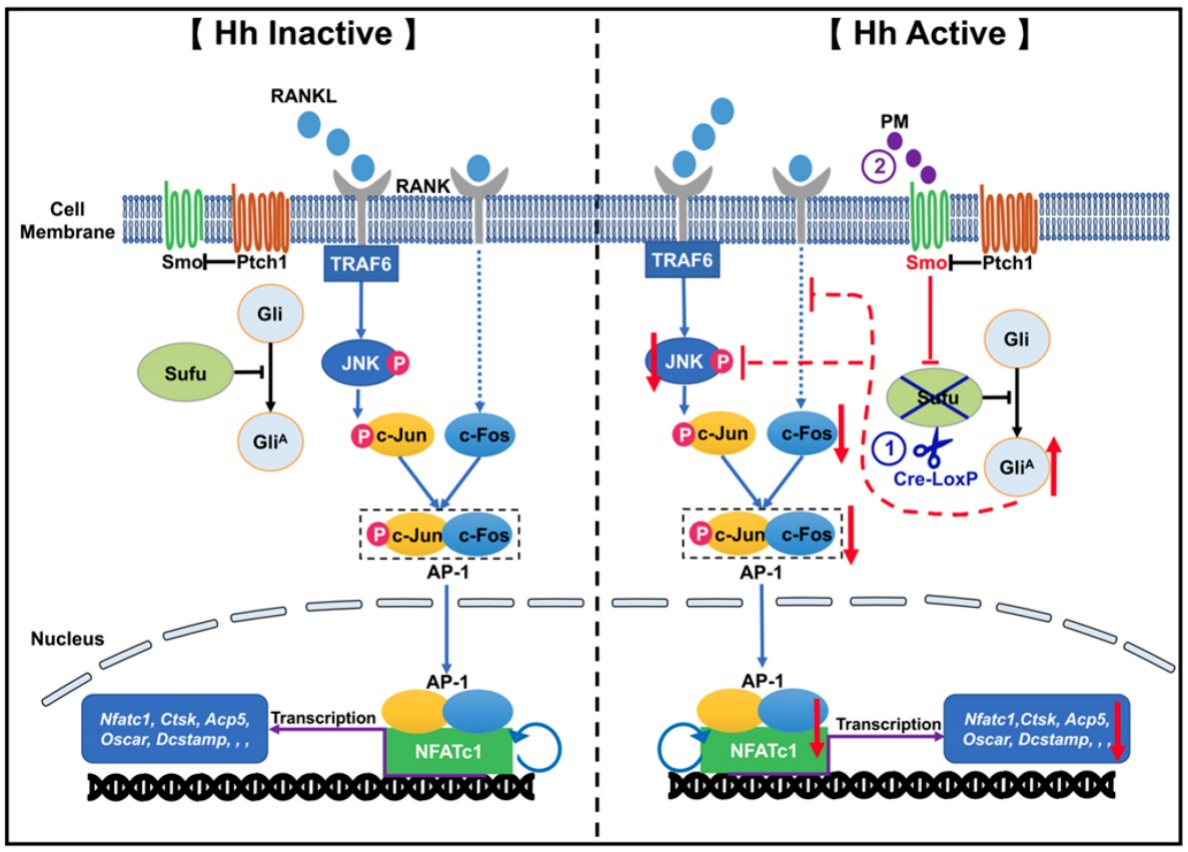
** A proposed model for the mechanism underlying the inhibitory effect of Hh signaling on osteoclastogenesis.** When Hh signaling is inactive (Left panel), binding of RANKL to RANK activates JNK signaling, which in turn phosphorylates and thus stimulates the activity of c-Jun. In addition, RANKL induces expression of c-Fos through an unknown mechanism. c-Jun forms the activator protein 1 (AP-1) complex with c-Fos to induce and amplify expression of *Nfatc1,* which further upregulates the transcription of osteoclast-specific genes such as *Ctsk*, *Acp5*, and *Oscar* and *Dcstamp*. Our results demonstrated for the first time that activation of Hh signaling by *Sufu* deletion or PM treatment impairs osteoclastogenesis via suppression of NFATc1 expression by inhibiting RANKL-indued actvation of JNK signaling and expression of c-Fos (Right panel).

In conclusion, by utilizing both genetic and pharmacological approaches to activate Hh signaling in osteoclast precursors, we proved that Hh signaling strongly inhibited RANKL-mediated osteoclast differentiation *in vitro* through suppression of the JNK/c-Fos/NFATc1 signaling cascade (**Figure 12**). Furthermore, we showed that activation of Hh signaling significantly reduced osteoclast formation and bone destruction in a murine calvarial model of Ti particle-induced osteolysis. To our knowledge, our study is the first to demonstrate that Hh pathway activation could be used as a promising therapeutic approach to prevent and treat PPO and other osteolytic diseases.

## Abbreviations

Acp5: acid phosphatase 5
AKT: protein kinase B
Atp6v0a3: ATPase H+ transporting V0 subunit A3
Atp6v0d2: ATPase H+ transporting V0 subunit D2
BMMs: bone marrow-derived macrophages
BSA: bovine serum albumin
BV/TV: bone volume/total tissue volume
CCK-8: cell counting kit-8
c-Fos: cellular protooncogene Fos
Ctsk: cathepsin K
DAPI: 4′,6-diamidino-2-phenylindole
Dcstamp: dendritic cell-specific transmembrane protein
ddH_2_O: double distilled water
DMSO: dimethyl sulfoxide
DPBS: Dulbecco's phosphate-buffered saline
EDTA: ethylenediaminetetraacetic acid
ERK: extracellular signal-regulated kinase
F-actin: fibrous actin
FBS: fetal bovine serum
FITC: fluorescein isothiocyanate
Gapdh: glyceraldehyde-3-phosphate dehydrogenase
Gli^A^: Gli activator
H: hour
H&E: hematoxylin and eosin
Hh: Hedgehog
JNK: c-Jun N-terminal kinase
MAPKs: mitogen-activated protein kinases
M-CSF: macrophage-colony stimulating factor
Min: minute
NFATc1: nuclear factor of activated T cells c1
NF-κB: nuclear factor kappa-B
Oscar: osteoclast-associated immunoglobulin-like receptor
P/S: penicillin/streptomycin
PFA: paraformaldehyde
PM: purmorphamine
PPO: periprosthetic osteolysis
Ptch1: patched1
qPCR: quantitative real-time polymerase chain reaction
RANK: receptor activator of nuclear factor-κB
RANKL: receptor activator of nuclear factor-κB ligand
RIPA buffer: radioimmunoprecipitation assay buffer
RT: room temperature
S: second
Smo: smoothened
Sufu: suppressor of fused
Ti: titanium
TJA: total joint arthroplasty
TRAP: tartrate-resistant acid phosphatase
α-MEM: α-modified Eagle's medium
μCT: micro-computed tomography
