# Supplementary material for: Genetic and pharmacological activation of Hedgehog signaling inhibits osteoclastogenesis and attenuates titanium particle-induced osteolysis partly through suppressing the JNK/c-Fos-NFATc1 cascade
Source: Theranostics. 2020 May 17;10(15):6638–60. doi: 10.7150/thno.44793 (PMC7295048; doi:10.7150/thno.44793)
Supplement: Supplementary file 1 — Supplementary figures and tables. [file thnov10p6638s1.pdf]

Table S1. Nucleotide sequences of primers used for qPCR analyses in this study.

Gene	Primer sequences (5′-3′)	
Sufu	Forward	GACGGTTCTAACCTGAGCGG
	Reverse	AGATGCTCCGGCTATCCTCT
Gli1	Forward	CCAAGCCAACTTTATGTCAGGG
	Reverse	AGCCCGCTTCTTTGTTAATTTGA
Ptch1	Forward	GCCTTGGCTGTGGGATTAAAG
	Reverse	CTTCTCCTATCTTCTGACGGGT
Nfatc1	Forward	CAACGCCCTGACCACCGATAG
	Reverse	GGCTGCCTTCCGTCTCATAGT
C-fos	Forward	TTGCTGATGCTCTTGACTGG
	Reverse	GGATTTGACTGGAGGTCTGC
Ctsk	Forward	ACGGAGGCATTGACTCTGAAGATG
	Reverse	GGAAGCACCAACGAGAGGAGAAAT
Dcstamp	Forward	TCCTCCATGAACAAACAGTTCCAA
	Reverse	AGACGTGGTTTAGGAATGCAGCTC
Acp5	Forward	TGTGGCCATCTTTATGCT
	Reverse	GTCATTTCTTTGGGGCTT
Oscar	Forward	CTGCTGGTAACGGATCAGCTCCCCAGA
	Reverse	CCAAGGAGCCAGAACCTTCGAAACT
Atp6v0a3	Forward	GGACCATATCCCTTTGGCATT
	Reverse	AAAGCTCAGGTGGTTCGTGG
Atp6v0d2	Forward	GTGAGACCTTGGAAGACCTGAA
	Reverse	GAGAAATGTGCTCAGGGGCT
Rn18s	Forward	CGGCTACCACATCCAAGGAA
	Reverse	GCTGGAATTACCGCGGCT

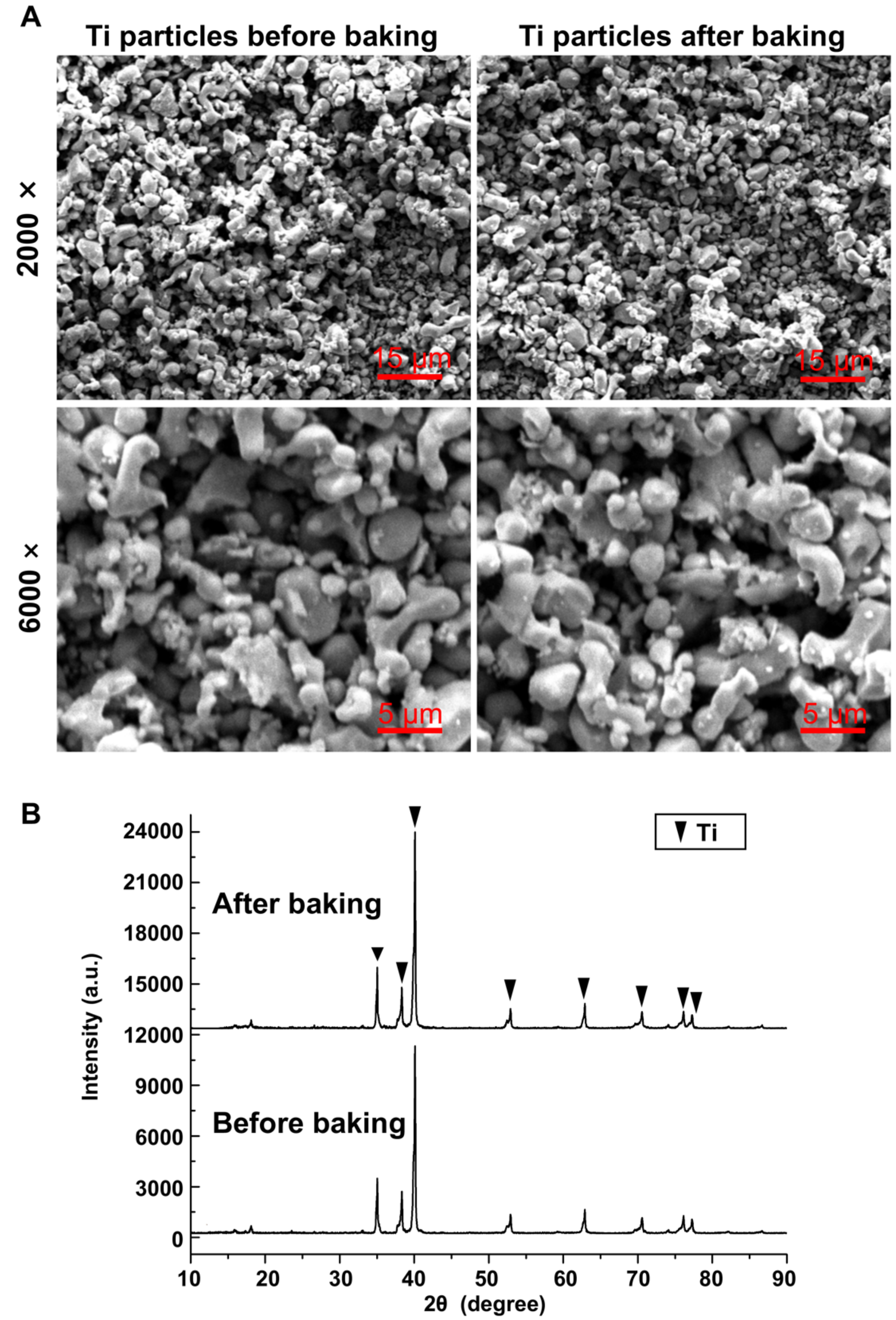

Figure S1. Characterization of Ti particles before and after 6 hours of baking at 180 ℃. (A) Representative SEM images showing the morphology of Ti particles before (left panels) and after (right panels) the baking treatment. **(B)** X-ray diffraction (XRD) patterns of Ti particles before (bottom) and after (top) 6 hours of baking at 180 ℃.

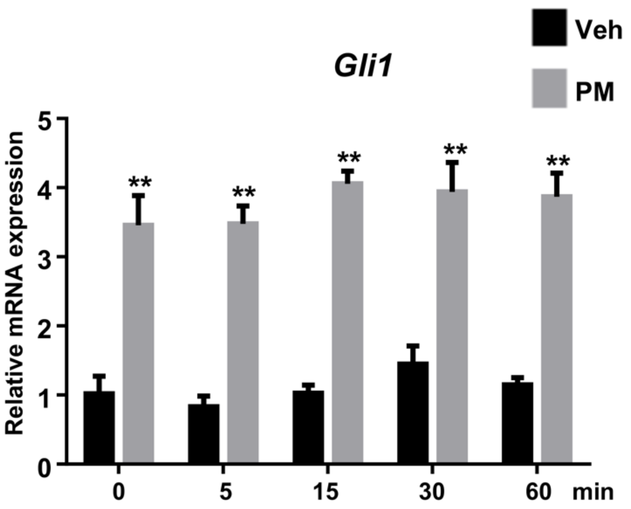

Figure S2. qPCR analysis of relative mRNA levels of *Gli1* in BMMs pretreated with vehicle or 2 μM PM for 4 h, and then stimulated with 50 ng/ml RANKL for indicated times in the absence or presence of 2 μM PM. *Gli1* expression was normalized by 18S ribosomal RNA. The relative changes in mRNA level were analyzed by $2^{-\Delta\Delta CT}$ method. All values were calculated from three independent biological replicates and presented as mean ± SD. ***P*<0.01, compared with vehicle-treated group.
